# Molecular characterization and functional analysis of buffalo prolactin (PRL) in mammary gland development and lactation

**DOI:** 10.5194/aab-68-703-2025

**Published:** 2025-12-03

**Authors:** Lige Huang, Xinyang Fan, Xiaohong Teng, Lindong Qian, Zhipeng Bao, Yongwang Miao

**Affiliations:** 1 Faculty of Animal Science and Technology, Yunnan Agricultural University, Kunming, Yunnan, China; 2 Animal Genetics and Breeding Institute, Yunnan Agricultural University, Kunming, Yunnan, China; 3 Faculty of Animal Husbandry and Veterinary Medicine, Yunnan Vocational College of Agriculture, Kunming, Yunnan, China; ℹ First correspondence author; ☆ Second correspondence author

## Abstract

This study investigated the molecular characteristics and functions of buffalo prolactin (PRL) in the lactation. The buffalo *PRL* gene's complete coding sequence (CDS) is 690 bp, encoding 229 amino acids, with structure and function highly consistent with other Bovidae species. *PRL* expression was significantly higher in the buffalo mammary gland during lactation compared to the non-lactating period, highlighting its crucial role in lactation. *PRL* overexpression in buffalo mammary epithelial cells (BuMECs) promotes cell proliferation and increases the casein secretion and triglyceride (TAG) accumulation. This occurs through the activation of the JAK2-STAT5 (cell differentiation, casein (*CSN*) gene transcription, and lipid factor transcription), PI3K-AKT-mTOR (cell growth, milk protein gene translation, and lipid factor activation), and mitogen-activated protein kinase (MAPK; cell proliferation and stress mitigation) signaling pathways. Specifically, *PRL* overexpression upregulated the mRNA expression of genes in these pathways, such as *JAK2*, *STAT5A*, *STAT5B*, *PI3K*, *AKT1*, *mTOR*, and *ERK2*, while decreasing *JNK1* and *P38*. Overexpression also increased the expression of cell cycle genes (*CDK2*, *CDK4*, *Cyclin D1*/*E1*), and enhanced cell viability. Furthermore, *PRL* overexpression led to increased expression of casein genes (*CSN2*, *CSN3*) and milk-fat-synthesis-related genes (*FASN*, *SCD*, *ACACA*, *PPARG*, *SREBF1*). Population genetic analysis identified five single-nucleotide polymorphisms (SNPs) in the buffalo *PRL* CDS, with c.34C > T and c.430T > C being non-synonymous substitutions that were predicted to affect protein function. This research provides a theoretical foundation for genetic interventions aimed at improving buffalo lactation traits.

## 1 Introduction

Prolactin (PRL) is a protein hormone synthesized and secreted by acidophils in the anterior pituitary gland. It is transported via blood circulation to target organs, where it regulates various metabolic processes in mammals (Binart et al., 2002; Goffin and Kelly, 1997). An early study established its role in inducing lactation in pseudopregnant female rabbits, leading to its naming as prolactin (Riddle et al., 1933). *PRL* belongs to the growth hormone/prolactin family, alongside growth hormone (*GH*) and placental prolactin (*PL*), all of which evolved from a common ancestral gene (Junnila et al., 2013). In mammals such as dairy cows, goats, sheep, and mice, PRL promotes mammary gland development, lactation, and the synthesis of milk fat and milk protein through the JAK2-STAT5 pathway (Bignon et al., 1999; Tian et al., 2015; Shi et al., 2016; Yamaji et al., 2013). Previous studies have confirmed a significant association between *PRL* polymorphisms and lactation traits, positioning PRL as a potential quantitative trait locus (QTL) or genetic marker for improving dairy cow lactation traits (Ghasemi et al., 2009; Dybus, 2002). Additionally, PRL can upregulate casein (*CSN*) mRNA expression in bovine mammary epithelial cells (Choi et al., 1988), while its inhibition leads to decreased milk production (Lacasse et al., 2011; Lollivier et al., 2015).

According to the NCBI database (https://www.ncbi.nlm.nih.gov/, last access: 11 September 2024), the buffalo *PRL* gene is located on BBU2 and consists of 5 exons and 4 introns. Two transcript variants have been annotated for this gene, with complete coding sequence (CDS) lengths of 690 and 693 bp, respectively. The bovine *PRL* gene also contains 5 exons and 4 introns, with a CDS length of 690 bp. A study reported that the first 30 amino acids at the N-terminus of bovine PRL protein constitute a signal peptide, with the mature protein comprising the remaining 199 amino acids (Cao et al., 2002). Furthermore, cloning of the swamp buffalo *PRL* coding region revealed a CDS length of 690 bp, encoding a protein of 229 amino acids, with the first 19 amino acids at the N-terminus forming the signal peptide (Du et al., 2009).

Buffalo are categorized into two types: swamp buffalo and river buffalo. Swamp buffalo are primarily used for plowing, whereas river buffalo are mainly utilized for milk production (El Nahas et al., 2013). Buffalo milk is nutritionally richer than cow's milk, containing higher levels of milk protein (58.2 % vs. 57.8 %, respectively), milk fat (70 vs. 41 g kg^−1^, respectively), and lactose (52.1 vs. 48.0 g kg^−1^, respectively) (Mota-Rojas et al., 2024). However, the milk yield of buffalo is comparatively low compared to dairy cows, partly attributed to a mammary gland with fewer total alveoli and epithelial cells (Mota-Rojas et al., 2024), alongside a delayed initiation of lactation (Prakash et al., 2005). To date, no study has systematically analyzed the synergistic regulatory mechanism of the buffalo *PRL* gene on the proliferation of mammary epithelial cells and milk synthesis. Furthermore, the polymorphism of the *PRL* coding region in Binglangjiang and Dehong buffalo remains unclear. Although the mechanism of PRL in mammalian lactation has been investigated, its specific mechanism in buffalo, and whether it is consistent with that in other mammals, particularly dairy cows, remains to be further elucidated. This gap is a major obstacle to understanding and improving buffalo milk production. Therefore, this study aims to analyze the molecular and functional characteristics of the buffalo *PRL* gene. We employed molecular cloning to identify the *PRL* CDS and analyzed its characteristics using bioinformatics and comparative genomics. Gene expression was quantified in various tissues and in buffalo mammary epithelial cells (BuMECs) via qPCR. Furthermore, we used a *PRL*-overexpressing BuMEC model to investigate its role in mammary development and the synthesis of milk protein and fat. Sanger sequencing was also used to identify genetic polymorphisms in the *PRL* gene between Binglangjiang and Dehong buffalo. This research provides a foundational understanding of buffalo PRL, offering a theoretical basis for strategies aimed at enhancing lactation and milk yield in this species.

## 2 Materials and methods

### 2.1 Sample collection

Throughout the experiment, efforts were made to minimize animal pain and suffering and reduce the number of experimental animals to a minimum. Binglangjiang buffalo (river type) and Dehong buffalo (swamp type) were obtained from core breeding farms in Tengchong and Dehong, Yunnan Province, respectively.

Tissue samples were collected from six healthy adult female Binglangjiang buffalo (approximately 4 years old, third parity), with three in peak lactation (approximately 60 d postpartum) and the other three in the dry period (approximately 60 d prepartum). After slaughter, tissue samples including heart, liver, spleen, lung, kidney, brain, cerebellum, rumen, small intestine, muscle, and mammary gland were immediately collected and stored in liquid nitrogen. These samples were used for total RNA extraction, gene cloning, and mRNA expression analysis. All buffalo were managed under identical feeding conditions.

In addition, blood samples were collected from 185 Binglangjiang buffalo and 200 Dehong buffalo for population variation analysis. Blood samples were placed in centrifuge tubes containing Tris-EDTA (TE) buffer and stored at -20 °C prior to DNA extraction. These buffalo were of similar age and parity and were unrelated.

### 2.2 Gene cloning and identification

Total RNA was extracted using the RNAiso Plus kit (Takara, Dalian, China) according to the manufacturer's instructions. RNA purity and concentration were assessed by measuring absorbance at 260/280 nm using a NanoDrop 2000 UV–Vis spectrophotometer (Thermo Fisher Scientific, Waltham, MA, USA). The integrity of 28S and 18S rRNA was further checked by agarose gel electrophoresis to ensure RNA quality. First-strand cDNA synthesis was performed by reverse-transcribing 1 µ g of RNA using Oligo(dT)18 primers (500 µ g mL^−1^) and a reverse transcriptase kit (Takara, Dalian, China) according to the manufacturer's instructions.

Primers for *PRL* CDS amplification (Table S1 in the Supplement) were designed using Primer Premier 5.0 (Lalitha, 2000) software based on the buffalo *PRL* mRNA sequence (EF054878.1) from the NCBI database. The 25 µ L PCR reaction mixture contained 0.8 µ L each of upstream and downstream primers (10 µ mol L^−1^), 15 µ L of 2 × Es Taq Master Mix (CWBIO, Beijing, China), 3 µ L of cDNA (50 ng µ L^−1^), and 5.4 µ L of ddH_2_O. PCR cycling conditions were 94 °C pre-denaturation for 2 min, followed by 35 cycles of 94 °C for 30 s, 60 °C for 40 s, and 72 °C for 50 s; a final extension at 72 °C for 5 min; and then termination at 4 °C. PCR products were visualized by 2 % agarose gel electrophoresis and purified using the TIANgel purification kit (Tiangen, Beijing, China). Purified products were ligated into the pMD-18T vector (Takara, Dalian, China) and transformed into *E. coli* DH5 α competent cells (TransGen Biotech, Beijing, China). Altogether, 15 positive clones were selected and sent to Shanghai Biotechnology Services Co., Ltd. (Shanghai, China) for bidirectional sequencing.

Sequence analysis and validation were performed using the SeqMan program in the Lasergene software package (DNAStar Inc., USA). Open reading frames (ORFs) were identified using the ORF Finder tool (https://www.ncbi.nlm.nih.gov/orffinder/, last access: 10 October 2024), and corresponding amino acid sequences were inferred using the EditSeq program (DNAStar Inc., USA). The obtained CDS was used as a query sequence for homologous search in the NCBI database (https://blast.ncbi.nlm.nih.gov/Blast.cgi, last access: 10 October 2024) using the BLAST program to confirm the target sequence.

### 2.3 Molecular characteristics and function prediction

The online software ProtParam (http://web.expasy.org/protparam, last access: 11 October 2024) was used to predict the molecular weight, isoelectric point, hydrophilicity, and other basic physicochemical characteristics of PRL. Signal peptides, transmembrane structures, functional modification sites, and subcellular localization were analyzed using SignalP-5.0 Server (https://services.healthtech.dtu.dk/services/SignalP-5.0/, last access: 11 October 2024), TMHMM-2.0 (https://services.healthtech.dtu.dk/services/TMHMM-2.0/, last access: 11 October 2024), ScanProsite (http://prosite.expasy.org/prosite.html, last access: 11 October 2024), and ProtComp 9.0 (http://www.softberry.com/berry.phtml?topic=protcompan&group=programs&subgroup=proloc, last access: 11 October 2024), respectively. Protein secondary structure prediction was performed using SOPMA (https://npsa-prabi.ibcp.fr/cgi-bin/npsa_automat.pl?page=/NPSA/npsa_sopma_f.html, last access: 11 October 2024), and three-dimensional structure modeling was performed using SWISS-MODEL (https://beta.swissmodel.expasy.org/, last access: 11 October 2024). Molecular functions and biological processes were analyzed using the DAVID (https://davidbioinformatics.nih.gov/, last access: 11 October 2024) online tool. Protein interaction networks were predicted using STRING (https://string-db.org/, last access: 11 October 2024), and parameters in STRING are default parameters (network type: full STRING network, required score: medium confidence (0.400), size cutoff: no more than 10 interactions).

To analyze sequence consistency, motif composition, and conserved domains, PRL sequences from 11 different species were retrieved and downloaded from the NCBI database (Table S2). The motif composition of PRL protein was analyzed using the online website MEME (http://meme-suite.org/tools/meme, last access: 12 October 2024), and conserved domains were identified using the NCBI Web CD-Search Tool (https://www.ncbi.nlm.nih.gov/Structure/bwrpsb/bwrpsb.cgi, last access: 12 October 2024). A phylogenetic tree of PRL amino acid sequences was constructed using the maximum likelihood method in MEGA7 (Kumar, et al., 2016) software based on the Jones–Taylor–Thornton (JTT) model, with bootstrap tests performed with 5000 replicates. PRL transcript data were obtained from genomic annotation feature files downloaded from the NCBI database. The complete transcriptional region structure of PRL was repaired using the GXF function in TBtools software and visualized using Gene Structure Display Server 2.0 (https://gsds.gao-lab.org/, last access: 12 October 2024).

### 2.4 Population variation detection

Polymorphisms in the *PRL* gene were detected by Sanger sequencing in Binglangjiang buffalo and Dehong buffalo populations. Genomic DNA was extracted using the traditional phenol/chloroform method (Russell and Sambrook, 2001). Primers for SNP detection were designed based on the buffalo *PRL* genomic sequence (NC_059158; Table S1) from the NCBI database. The 25 µ L PCR reaction mixture included 0.8 µ L of upstream and downstream primers (10 µ mol L^−1^), 15 µ L of 2 × Es Taq Master Mix (CWBIO, Beijing, China), 3 µ L of DNA template (50 ng µ L^−1^), and 5.4 µ L of ddH_2_O. PCR conditions were 94 °C pre-denaturation for 2 min; 35 cycles of 94 °C for 30 s, 60 °C for 40 s, and 72 °C for 50 s; and a final extension at 72 °C for 5 min, with the reaction terminated at 4 °C. The target PCR products, detected by 1 % agarose gel electrophoresis, were sent to Shanghai Biotechnology Services Co., Ltd. (Shanghai, China) for bidirectional sequencing.

The obtained sequences were proofread and edited, and SNPs were output using SeqMan software in the Lasergene 7 software package (DNAStar Inc., USA). Genotypes, gene frequency estimation, and Hardy–Weinberg equilibrium tests were performed using Popgene (Yeh, 1997) software. The haplotype of SNPs was inferred using PHASE (Gowri-Shankar and Jow, 2006) software. PROVEAN (Choi and Chan, 2015) software was used to predict the effect of non-synonymous amino acid substitutions on protein function.

### 2.5 Cell culture

BuMECs were isolated from mammary gland tissue of lactating buffalo (60 d postpartum), and purified based on the differential sensitivity of the cells to trypsin digestion as previously described by our group (Fan et al., 2020). The BuMECs identified by cytokeratin 18 (Sigma, St. Louis, MO, USA) were cultured in DMEM (Gibco, Carlsbad, CA, USA) supplemented with 5 µ g mL^−1^ hydrocortisone (Sigma, St. Louis, MO, USA), 5 µ g mL^−1^ insulin (Sigma, St. Louis, MO, USA), 1 µ g mL^−1^ epidermal growth factor (Sigma, St. Louis, MO, USA), 2 % penicillin/streptomycin (Gibco, Carlsbad, CA, USA), and 10 % fetal bovine serum (Gibco, Carlsbad, CA, USA). Before the start of the experiment, BuMECs were cultured in the above medium containing 3 µ g mL^−1^ prolactin (Sigma, St. Louis, MO, USA) for 48 h to induce lactation (Matusik and Rosen, 1978; Song et al., 2021; Liu et al., 2023; Gao et al., 2024). Subsequently, the medium was replaced with hormone- and growth-factor-free DMEM supplemented with 10 % fetal bovine serum for the experiment.

### 2.6 Overexpression of *PRL* in BuMECs

Gene function was detected by the transient transfection of cells. Based on the *PRL* sequence obtained in this study, primers for plasmid construction (B1; Table S1) were designed using Primer Premier 5.0 software. The PCR reaction system and conditions were the same as those used for gene cloning. PCR products and pEGFP-N1 vector were digested with HindIII and EcoRI restriction endonucleases (Takara, Dalian, China), purified using a DNA gel extraction kit (Tiangen, Beijing, China), and ligated with T4 DNA ligase (Takara, Dalian, China). The constructed plasmid (pEGFP-N1-*PRL*) was sequenced to verify the correct insertion of the target sequence. In total, 10 kanamycin-resistant positive clones were selected for bidirectional sequencing (with a sequencing coverage of ≥3× ). Of these clones, 8 showed sequences entirely consistent with the expected *PRL* CDS. One correct clone was selected, and the EndoFree Plasmid Kit (Tiangen, Beijing, China) was used to extract the plasmid for subsequent transfection, with a final concentration of ≥1 µ g µ L^−1^.

When cells reached approximately 70 %–80 % confluence in six-well plates, 3 µ g of pEGFP-N1-*PRL* plasmid or 3 µ g of pEGFP-N1 empty vector was transiently transfected into BuMECs using TransIntro™ EL transfection reagent (TransGen Biotech, Beijing, China) according to the manufacturer's instructions. Simultaneously, a blank control group consisting of untreated BuMECs was included. After 48 h of transfection, cell samples were collected for RNA extraction and for casein and triglyceride (TAG) detection. All experiments were performed in triplicate.

### 2.7 Real-time fluorescence quantitative PCR (RT-qPCR)

Gene expression levels in various buffalo tissues and BuMECs were detected by RT-qPCR. Total RNA extraction from tissues and cells was performed as described in Sect. 2.2. mRNA expression level analysis was performed using an IQ5 RT-PCR instrument (Bio-Rad, CA, USA) and SYBR Premix Ex Taq (Takara, Dalian, China). Each qPCR reaction (total volume 20 µ L) contained 10 µ L of SYBR Premix Ex Taq, 0.8 µ L each of upstream and downstream primers (10 µ mol L^−1^), 2 µ L of cDNA template (200 ng µ L^−1^), and 6.4 µ L of ddH_2_O. qPCR conditions were 95 °C pre-denaturation for 3 min, followed by 40 cycles of 95 °C for 5 s, 60 °C for 20 s, and 72 °C for 30 s. Melt curve analysis was performed by increasing the temperature by 0.5 °C every 5 s within the temperature range of 65 to 95 °C. The specificity of PCR products was determined by melt curve analysis and sequencing. The efficiency of amplification was determined using LinRegPCR (Table S1). The relative mRNA expression level of the target gene was normalized to the geometric mean of the expression levels of three housekeeping genes (*ACTB*, *GAPDH*, and *RPS23*). All reactions were repeated three times to ensure data reliability. Primer information is detailed in Table S1.

### 2.8 Detection of TAG and casein content

The intracellular TAG and total casein content in BuMECs were detected using corresponding kits according to the manufacturer's instructions. Intracellular TAG content was determined using a tissue/cell triglyceride assay kit (Pulilai, Beijing, China). Total cell protein concentration was determined using a BCA kit (Beyotime, Shanghai, China). TAG concentration was normalized to total protein content and reported as µ M g^−1^ protein. Total cell casein concentration was determined using a bovine casein ELISA kit (Enzyme-linked, Shanghai, China).

### 2.9 Cell viability assay

Cell viability was assessed using a CCK-8 cell counting kit (Shangwei, Shenzhen, China) in 96-well plates (2 × 10^3^ cells per well). BuMECs were seeded in 96-well plates, and when cell confluence reached 70 %–80 %, pEGFP-N1-*PRL* plasmid or pEGFP-N1 empty vector was transfected into BuMECs in quintuplicate. Subsequently, 10 µ L of CCK-8 solution was added to 100 µ L of cell suspension and incubated for 2 h. Absorbance at 450 nm was measured in triplicate using a microplate reader (Varioskan Lux, Thermo Scientific, USA).

### 2.10 Data analysis

All data were analyzed and visualized using GraphPad Prism 5 software (GraphPad software Inc., La Jolla, CA, USA). Experimental results are presented as the mean ± standard error of the mean (mean ± SEM) of all independent experiments. For qPCR data, the 2^−ΔΔ*C* *t*^ method was used for quantitative analysis of relative expression. A Student's t test was specifically used for pairwise comparisons between two experimental groups, and p< 0.05 was considered statistically significant, while p> 0.05 indicated no significant difference.

## 3 Results

### 3.1 Cloning and identification of buffalo *PRL* gene

Using buffalo mammary gland cDNA as a template, a specific fragment with a length of 750 bp was cloned, consistent with the expected results (Fig. S1 in the Supplement). Sequence analysis of the cloned product using the ORF program determined that its CDS length was 690 bp, encoding a polypeptide composed of 229 amino acid residues (Fig. S2). A homologous search in the NCBI database using this CDS as a query sequence showed 99.9 % sequence identity with the buffalo *PRL* gene (NM_001290885.1) already included in the database. Based on this high sequence similarity, the cloned gene was identified as the buffalo *PRL* gene. The A, G, T, and C base contents in the buffalo *PRL* CDS were 25.07 %, 23.77 %, 23.33 %, and 27.83 %, respectively, with a G + C content of 57.27 %.

### 3.2 Transcriptional region structure of *PRL*

**Figure 1:**
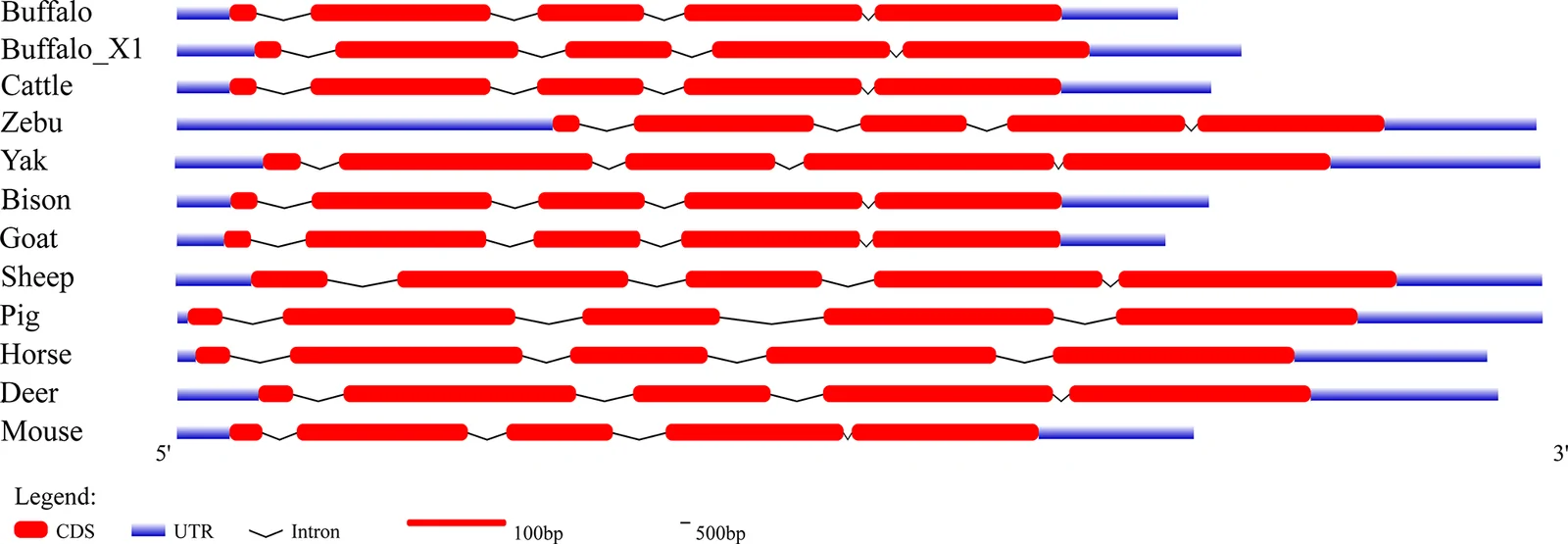
Transcriptional region structure of the *PRL* gene in buffalo and 10 other species.

To further elucidate the structural characteristics of the buffalo *PRL* gene transcriptional region, this study performed a comprehensive comparative analysis of the complete *PRL* mRNA sequences from buffalo and 10 other species (Table 1 and Fig. 1). In the NCBI database, two mRNA sequences for the buffalo *PRL* gene exist: NM_001290885.1 and XM_006072861.4 (buffalo_X1). CDS alignment revealed a one-codon difference in the exon 2 region between the two. Further analysis of the CDS lengths in Bovidae species showed that, except for sheep, the CDS length of buffalo was consistent with other Bovidae species. Additionally, the CDS length of buffalo was similar to that of other non-Bovidae mammals but differed from that of mice. Due to sequence differences in exon 1 and exon 5, the untranslated region (UTR) of buffalo *PRL* mRNA was inconsistent with the UTRs of other Bovidae species and other non-Bovidae mammals. Nevertheless, the transcriptional region structure of the buffalo *PRL* gene was still highly similar to that of other mammals. Specifically, the *PRL* gene in both buffalo and other Bovidae species or common non-Bovidae mammals contained 5 exons and 4 introns. This conserved gene structure further supports the importance of the *PRL* gene in maintaining its function during evolution.

**Table 1:** Information on *PRL* transcriptional region structures analyzed in this study.

Species	Transcript ID	Length/bp							
		5' UTR	Exon1	Exon2	Exon3	Exon4	Exon5	3' UTR	CDS
Buffalo	NM_001290885.1	54	82	182	108	180	307	118	687
Buffalo_X1	XM_006072861.4	79	107	185	108	180	343	154	690
Cattle	NM_173953.2	54	82	182	108	180	341	152	687
Zebu	XM_019986186.1	379	407	182	108	180	343	154	687
Yak	XM_005894272.2	64	92	182	108	180	343	154	687
Bison	XM_010845567.1	55	83	182	108	180	338	149	687
Sheep	NM_001009306.1	60	121	182	108	180	334	145	695
Goat	NM_001285547.1	48	76	182	108	180	295	106	690
Pig	NM_213926.1	9	37	182	108	180	334	145	687
Horse	NM_001081896.1	15	43	182	108	180	340	151	687
Deer	XM_043908398.1	64	92	182	108	180	336	147	687
Mouse	NM_011164.2	54	88	173	108	180	346	157	684

Note: CDS lengths are shown without the stop codon.

### 3.3 Molecular characteristics and phylogenetic relationships of PRL

**Figure 2:**
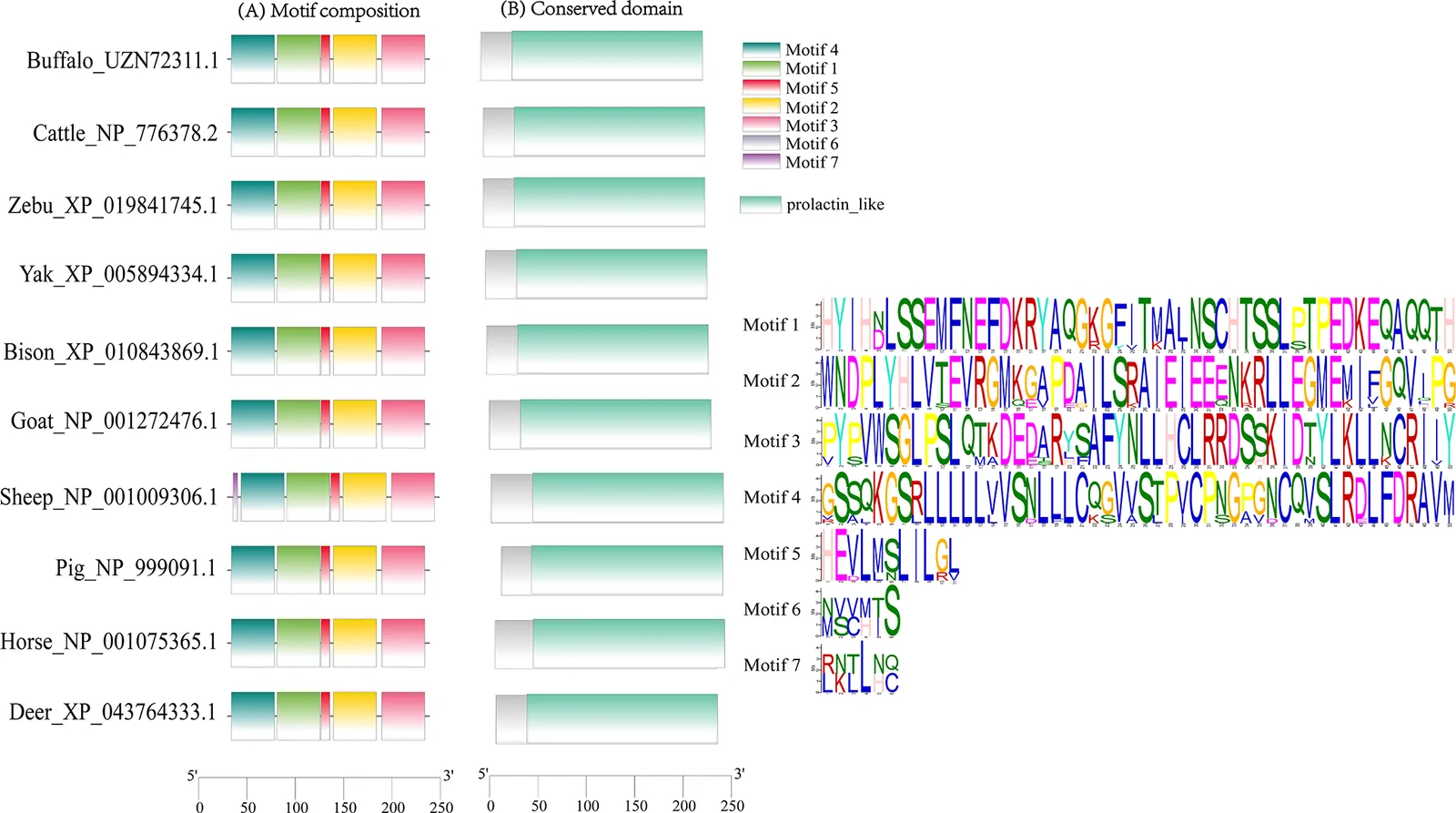
Motif composition and conserved domain of PRLs in buffalo and other mammals.

**Figure 3:**
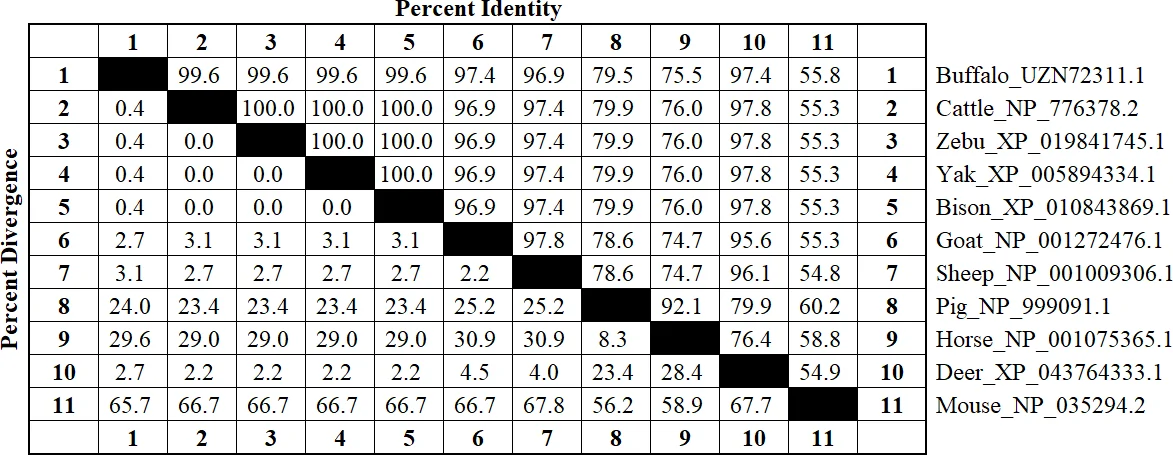
Amino acid sequence identity of PRL in buffalo and 10 other species.

**Figure 4:**
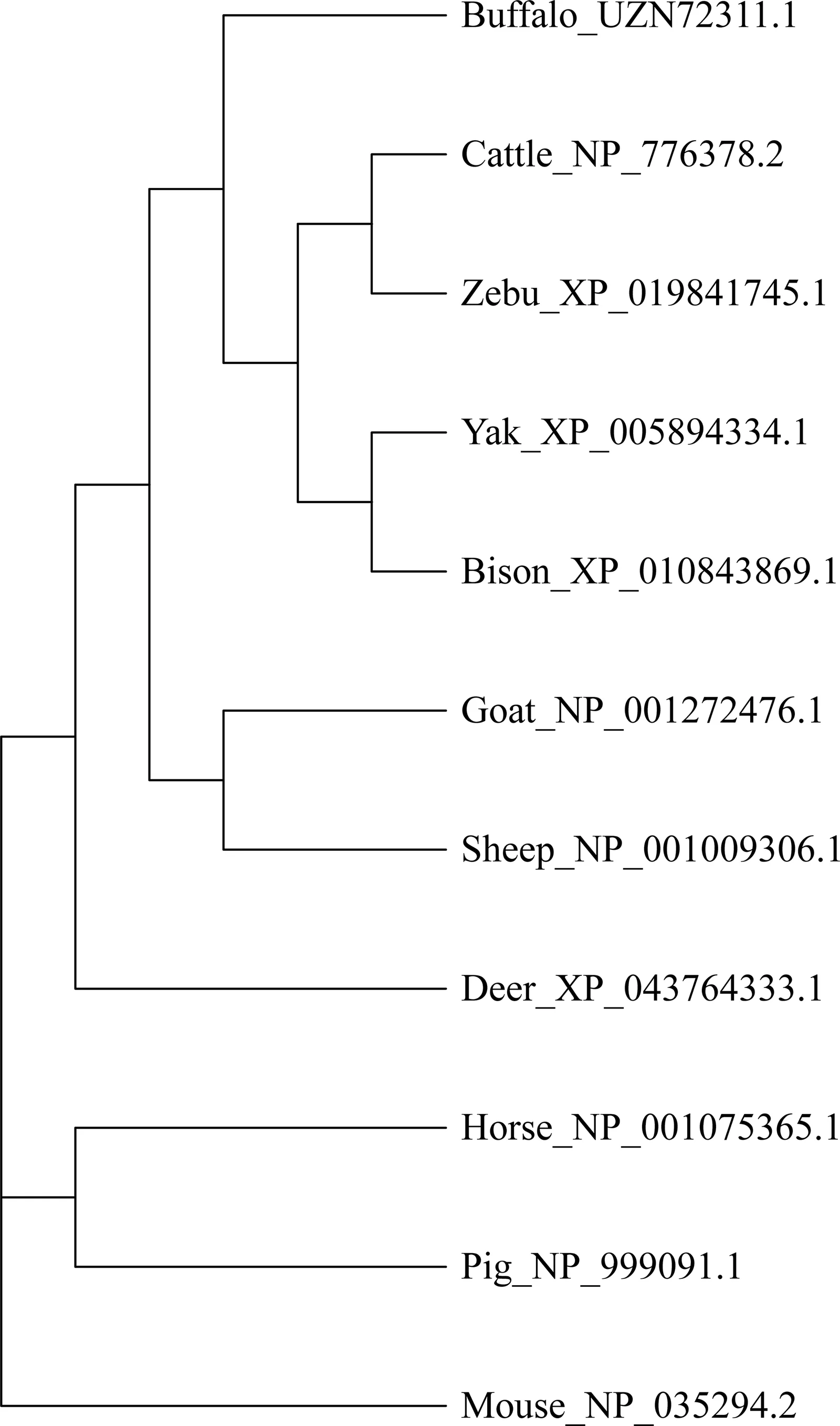
Phylogenetic relationships of PRLs in buffalo and other mammals.

This study analyzed the molecular characteristics and evolutionary relationships of PRL in buffalo and other mammals. Buffalo PRL protein contains an N-terminal signal peptide (AA1-30, score 9.64) and five types of functional modification sites (Fig. S3), and its physicochemical characteristics are highly similar to those of Bovidae species (Fig. S4 and Table S3). Seven motifs and one conserved domain were identified in mammalian PRLs (Fig. 2). Buffalo and other Bovidae species, as well as other mammals, share motifs 1–6, while sheep PRL has an additional motif 7. Mammalian PRLs all contain a prolactin-like domain, which is composed of motifs 1–6. Amino acid sequence alignment showed that buffalo PRL has 96.9 %–100 % identity with Bovidae PRLs (Fig. 3). Phylogenetic analysis showed that buffalo clustered first with *Bos* species and then with *Ovis* species (Fig. 4). Secondary structure analysis and homology modeling showed that the secondary and three-dimensional structures of buffalo PRL are extremely similar to those of other Bovidae PRLs (Figs. S5, S6, Table S4). These results indicate that PRL in buffalo and other mammals is evolutionarily conserved and highly functionally consistent, especially with other Bovidae species.

### 3.4 Molecular function, biological processes, and protein interactions

**Figure 5:**
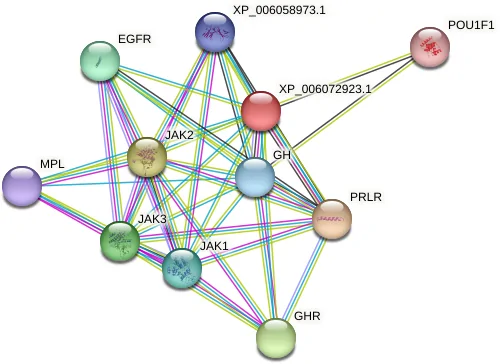
Predicted interacting proteins of buffalo PRL. The color of the lines represents different modes of predicted interaction: light green represents text mining, red lines represent gene fusion, purple represents experimental determination, and cyan represents data from databases.

Buffalo PRL is an extracellular secreted protein, and its molecular function is primarily to bind to the prolactin receptor (PRLR), thereby participating in biological processes such as mammary gland development, lactation, and positive regulation of fatty acid synthesis through the PI3K-AKT and JAK-STAT signaling pathways. The analysis predicted that buffalo PRL may interact with 10 proteins (Fig. 5), including GH, growth hormone receptor (GHR), PRLR, leptin receptor (LEPR), epidermal growth factor receptor (EGFR), myeloproliferative leukemia protein (MPL), Janus kinases (JAK1–3), and POU class 1 homeobox 1 (POU1F1). Its interacting proteins also participate in the PI3K-AKT and JAK-STAT signaling pathways. This indicates that buffalo PRL, as the core, collaborates with multiple interacting proteins to jointly regulate important physiological activities such as mammary gland development and lactation by participating in key signaling pathways.

**Figure 6:**
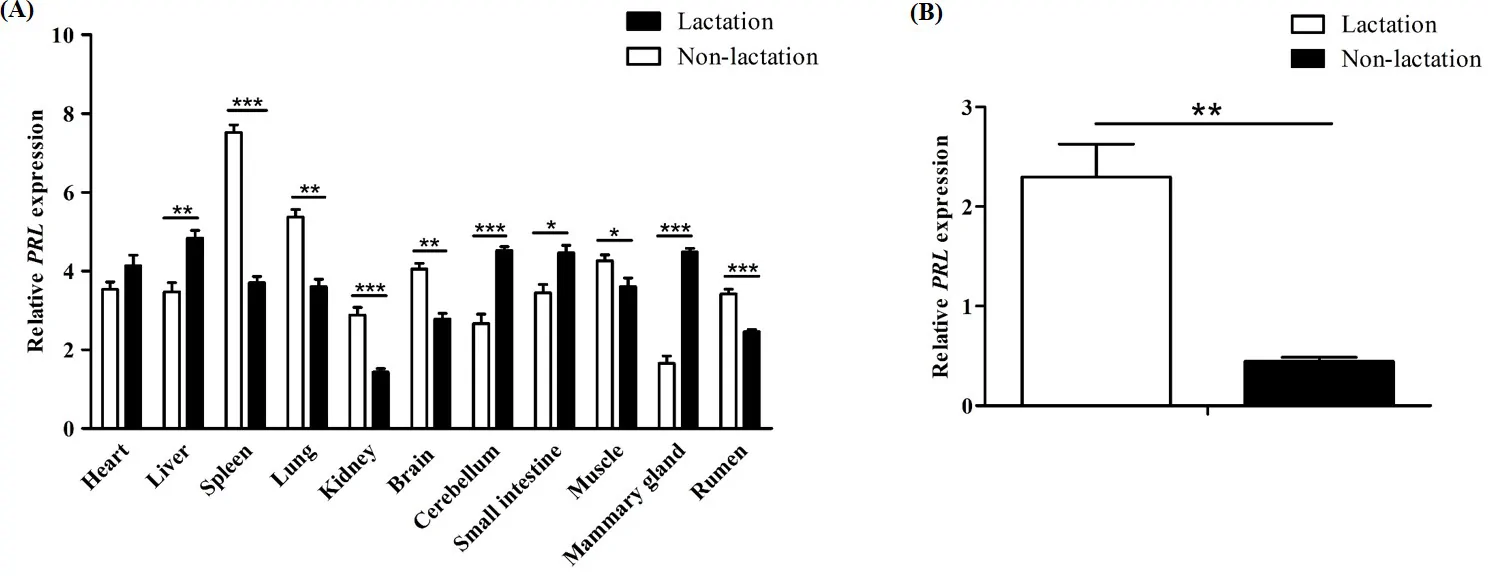
Differential expression of the *PRL* gene in buffalo. **(A)** Differential tissue expression of buffalo *PRL* during lactation and non-lactation; **(B)** expression of *PRL* in lactating and non-lactating BuMECs. Data represent the mean ± SEM of three independent experiments. ^*^ p< 0.05; ^**^ p< 0.01; ^***^ p< 0.001.

### 3.5 Differential gene expression

The qPCR results showed that *PRL* was expressed in all tested tissues (Fig. 6A). During lactation, *PRL* mRNA was highly expressed in the heart, liver, cerebellum, small intestine, and mammary gland, while it was lowly expressed in the kidney, brain, and rumen. During the non-lactating period, *PRL* mRNA was highly expressed in the spleen, lung, brain, and muscle, while it was lowly expressed in the mammary gland, cerebellum, and kidney. Notably, the mRNA abundance of *PRL* in mammary gland tissue was significantly higher during lactation than in the non-lactating period ( p< 0.001). Furthermore, the mRNA abundance of *PRL* in lactating and non-lactating BuMECs was also detected (Fig. 6B). The results showed that the expression abundance of *PRL* was significantly higher in the lactating state than in the non-lactating state ( p< 0.01).

**Figure 7:**
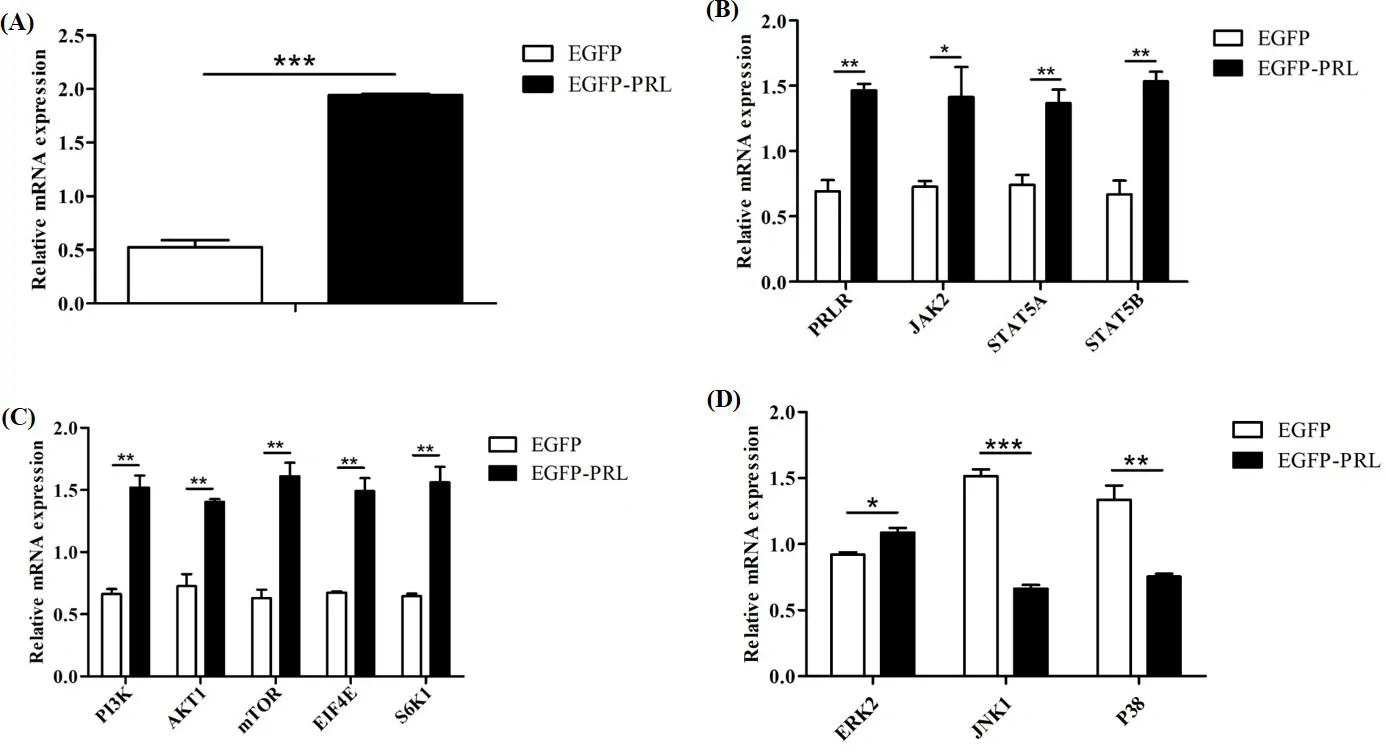
Effects of *PRL* overexpression on mRNA abundance of genes in JAK2-STAT5, mTOR, and MAPK pathways. **(A)** Overexpression efficiency of *PRL*, **(B)** changes in gene expression in the JAK2-STAT5 pathway after *PRL* overexpression, **(C)** changes in gene expression in the mTOR pathway due to *PRL* overexpression, **(D)** changes in gene expression in the MAPK pathway after *PRL* overexpression. Data are expressed as the mean ± SEM of three independent experiments. ^*^ p< 0.05; ^**^ p< 0.01; ^***^ p< 0.001.

### 3.6 PRL positively regulates JAK2-STAT5, mTOR, and MAPK signaling pathways

To elucidate the regulatory role of PRL in mammary gland development and milk protein and milk fat synthesis, we detected the expression levels of key genes involved in these processes after *PRL* overexpression in BuMECs (Fig. 7). These genes included upstream genes of the JAK2-STAT5 pathway (*PRLR*, *JAK2*,* STAT5A*, *STAT5B*), genes of the mTOR pathway (*PI3K*, *AKT1*, *mTOR*) and downstream genes (*EIF4E*, *S6K1*), and genes in the mitogen-activated protein kinase (MAPK) pathway (*ERK2*, *JNK1*, *P38*). Compared to the control group (EGFP), the mRNA abundance of *PRL* in EGFP-*PRL* infected BuMECs significantly increased by 73 % ( p< 0.001). *PRL* overexpression significantly increased the mRNA abundance of *PRLR* ( p< 0.01), *JAK2* ( p< 0.05), *STAT5A* ( p< 0.01), *STAT5B* ( p< 0.01), *PI3K* ( p< 0.01), *AKT1* ( p< 0.01), *mTOR* ( p< 0.01), *EIF4E* ( p< 0.01), *S6K1* ( p< 0.01), and *ERK2* ( p< 0.05), while significantly decreasing the mRNA abundance of *JNK1* ( p< 0.001) and *P38* ( p< 0.01).

**Figure 8:**
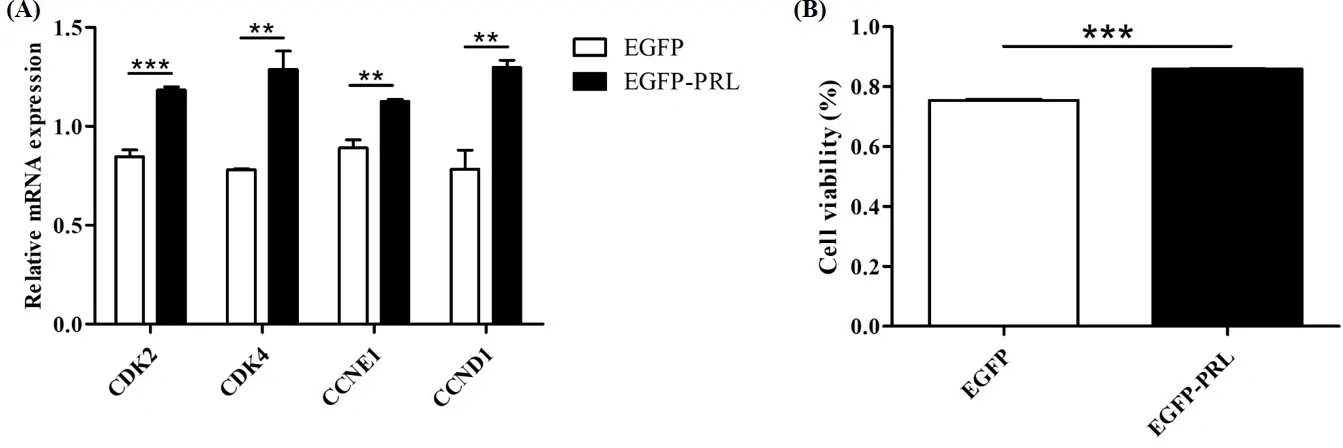
Effects of *PRL* overexpression on cell proliferation. **(A)** Effects of *PRL* overexpression on the expression of cell-cycle-related genes; **(B)** effects of *PRL* overexpression on cell viability. Data are the mean ± SEM of three independent experiments. ^*^ p< 0.05; ^**^ p< 0.01; ^***^ p< 0.001.

### 3.7 PRL promotes BuMECs proliferation

To investigate the role of PRL in mammary gland development, the expression abundance of cell-cycle-related genes and the viability of BuMECs were detected by qPCR and CCK-8 assay, respectively, after *PRL* overexpression. The results showed that *PRL* overexpression significantly upregulated the expression levels of cell cycle genes *CDK2* ( p< 0.001), *CDK4* ( p< 0.01), *Cyclin D1* ( p< 0.01), and *Cyclin E1* ( p< 0.01; Fig. 8A) and significantly increased cell viability ( p< 0.001; Fig. 8B).

**Figure 9:**
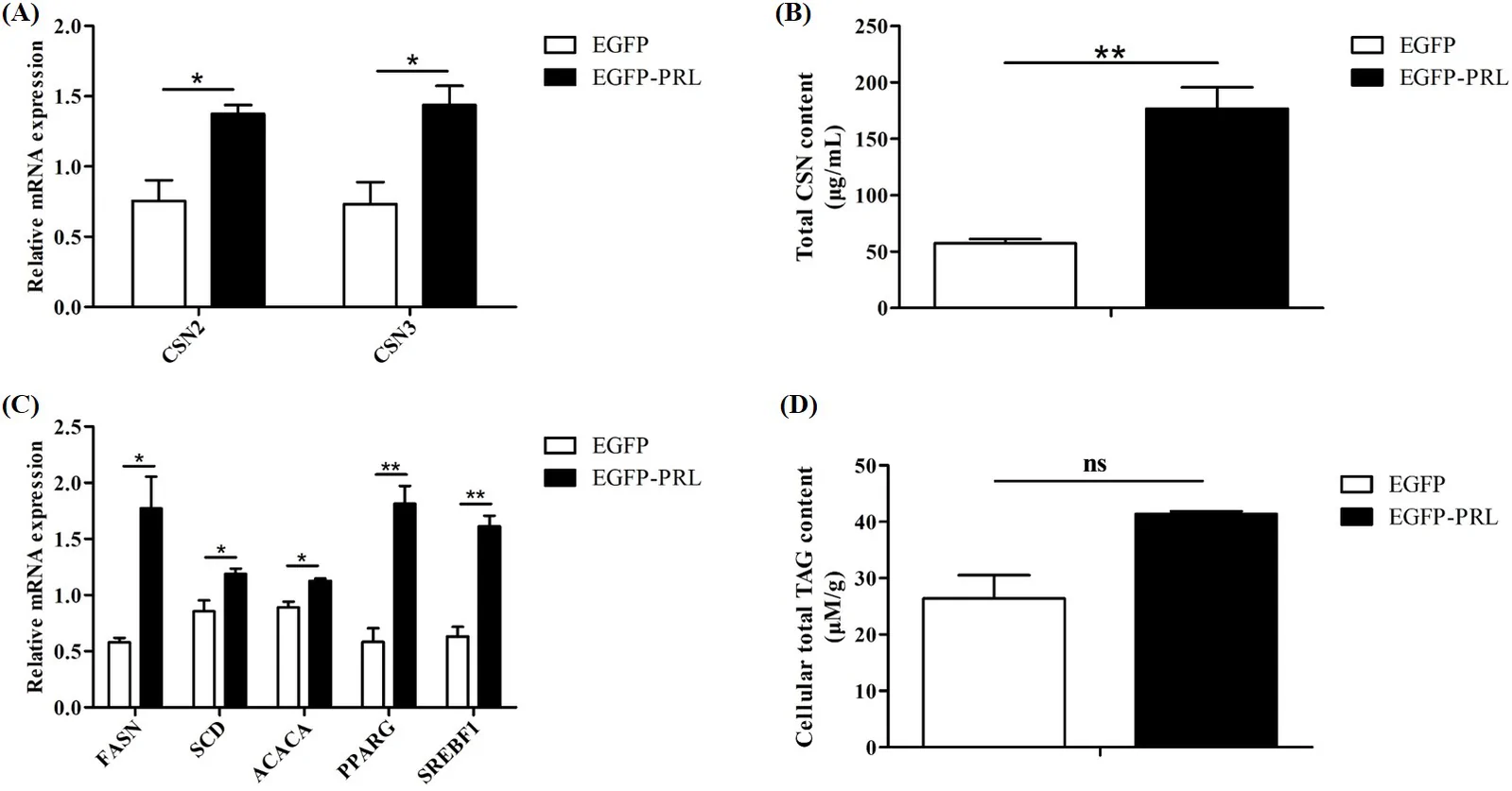
Effects of *PRL* overexpression on milk protein and milk fat synthesis. **(A)** Expression of casein gene after *PRL* overexpression, **(B, D)** changes in casein or TAG content in BuMECs after *PRL* overexpression, **(C)** effects of *PRL* overexpression on genes related to milk fat synthesis. Data are the mean ± SEM of three independent experiments. ^*^ p< 0.05; ^**^ p< 0.01; ns, p> 0.05.

### 3.8 PRL promotes milk protein and milk fat synthesis in BuMECs

To investigate the role of PRL in buffalo milk protein and milk fat synthesis, recombinant plasmid EGFP-*PRL* was transfected into BuMECs. After 48 h, the expression abundance of casein genes and casein content, along with the expression abundance of milk fat synthesis-related genes and TAG content, was detected. Overexpression of *PRL* significantly increased the expression levels of casein genes ( p< 0.05; Fig. 9A) and casein content ( p< 0.01; Fig. 9B) in BuMECs. In addition, the gene expression of *FASN* ( p< 0.05), *SCD* ( p< 0.05), *ACACA* ( p< 0.05), *PPARG* ( p< 0.01), and *SREBF1* ( p< 0.01) related to milk fat in BuMECs was significantly increased (Fig. 9C), and an increasing trend in intracellular TAG content was observed, but the difference was not statistically significant ( p> 0.05; Fig. 9D).

### 3.9 Population variation and sequence differences of haplotypes

Five SNPs were identified in the buffalo *PRL* CDS (Table 2). Among them, c.427C > T and c.430T > C were shared by the two buffalo types, c.34C > T and c.606C > T were only found in river buffalo, and c.192G > A was only found in swamp buffalo. In river buffalo, c.192 was homozygous for the GG genotype; the c.427 locus tended to be homozygous (CC genotype). In swamp buffalo, c.34 and c.606 were homozygous for the CC genotype; the c.430 locus tended to be homozygous (TT genotype). A Hardy–Weinberg equilibrium test showed that SNP430 was in disequilibrium in swamp buffalo ( p< 0.05), and the polymorphic information content (PIC) for this locus was 0.0905, indicating a low level of genetic diversity. There were significant differences in homozygosity and heterozygosity between the shared SNP sites (c.427C > T and c.430T > C) in the two buffalo types. Nucleotide sequence analysis showed that c.192G > A, c.427C > T, and c.606C > T were synonymous substitutions and had no effect on codon usage bias (Table S5). c.34C > T and c.430T > C were non-synonymous substitutions, leading to p.R12C and p.S144P amino acid changes, but prediction showed that they had no effect on buffalo PRL function.

**Figure 10:**
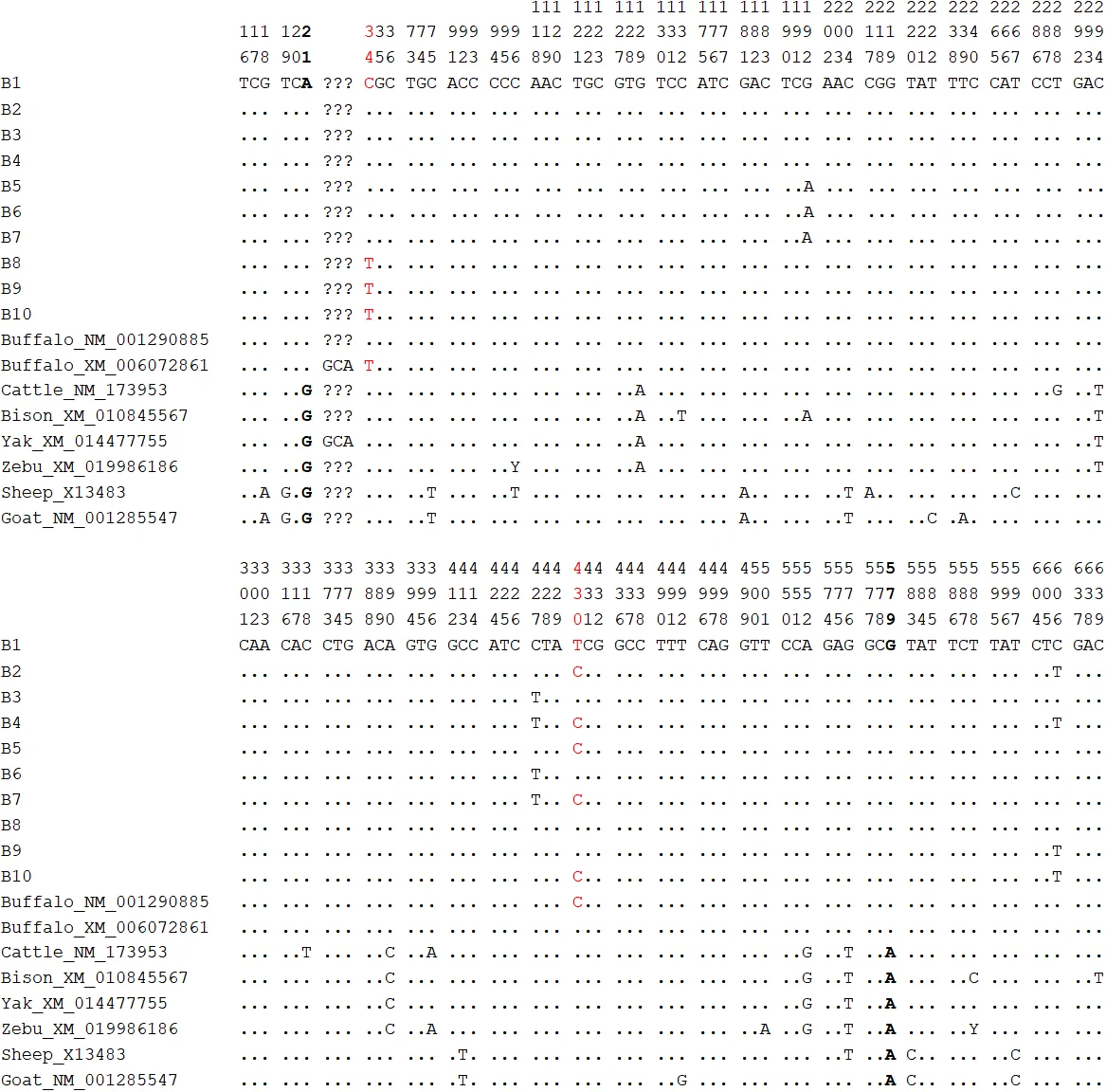
Nucleotide difference sites of buffalo *PRL* haplotypes and other Bovidae *PRL* sequences. Numbers indicate positions in the coding region, dots (.) indicate consistency with B1, nucleotide substitutions are indicated by different letters, and missing information is marked with a question mark (?). The same applies below.

Based on these 5 SNPs, 10 haplotypes (B1–B10; Table S6) were defined. B1 was common to river and swamp buffalo; B2, B4, B8, B9, and B10 were found only in river buffalo; and B3, B5, B6, and B7 were found only in swamp buffalo. Among them, haplotype B1 had the highest frequency and was the major haplotype. Comparative analysis was performed between the buffalo *PRL* haplotype sequences from this study and published homologous *PRL* sequences of Bovidae species from the NCBI database (only one sequence was taken for the same species if identical; Figs. 10 and S7). Buffalo (NM_001290885) and yak (XM_014477755) had one more codon compared to B1. The buffalo *PRL* sequence had 6 nucleotide difference sites (c.21, c.129, c.294, c.390, c.552, c.579) with *Bos* species but no difference in encoded amino acids. There were 10 nucleotide difference sites (c.18, c.19, c.21, c.75, c.181, c.204, c.413, c.579, c.583, c.597) with *Ovis* species, among which c.19, c.413, and c.583 led to changes in encoded amino acids (p.S7A, p.A138V, p.Y195H). Notably, c.21 and c.579 were common nucleotide difference sites between buffalo and *Bos* and *Ovis* species.

**Table 2:** Population variation analysis of buffalo *PRL* CDS.

Population	SNP	Genotype	Frequency	Allele	Frequency	Expected	P	Polymorphic
		and	(%)		(%)	hetero-	value	information
		number				zygosity		content
		of						
		individuals						
River		CC (102)	0.551	C	0.7405	0.4864	0.8056	0.3105
buffalo	c.34C > T	CT (70)	0.379	T	0.2595			
		TT (13)	0.070					
	c.192G > A	GG (185)	1.000	G	1.0000	0.0000	–	–
		GA (0)	0.000	A	0.0000			
		AA (0)	0.000					
	c.427C > T	CC (184)	0.995	C	0.9973	0.0054	1.0000	0.0054
		CT (1)	0.005	T	0.0027			
		TT (0)	0.000					
		CC (4)	0.022	C	0.1405			
	c.430T > C	CT (44)	0.238	T	0.8595	0.2422	0.8034	0.2124
		TT (137)	0.740					
		CC (102)	0.551	C	0.7405			
	c.606C > T	CT (70)	0.379	T	0.2595	0.3853	0.8056	0.3105
		TT (13)	0.070					
Swamp	c.34C > T	CC (200)	1.000	C	1.0000	0.0000	–	–
buffalo		CT (0)	0.000	T	0.0000			
		TT (0)	0.000					
	c.192G > A	GG (129)	0.645	G	0.7950	0.3268	0.2442	0.2728
		GA (60)	0.300	A	0.2050			
		AA (11)	0.055					
	c.427C > T	CC (78)	0.390	C	0.6125	0.4759	0.3575	0.3620
		CT (89)	0.445	T	0.3875			
		TT (33)	0.165					
	c.430T > C	CC (2)	0.010	C	0.0500	0.0952	0.0204	0.0905
		CT (16)	0.080	T	0.9500			
		TT (182)	0.910					
	c.606C > T	CC (200)	1.000	C	1.0000	0.0000	–	–
		CT (0)	0.000	T	0.0000			
		TT (0)	0.000					

## 4 Discussion

This study demonstrates that PRL promotes mammary epithelial cell proliferation and milk synthesis through coordinated activation of three key signaling pathways: JAK2-STAT5 (critical for cell differentiation and transcriptional regulation of milk protein and lipid synthesis genes), PI3K-AKT-mTOR (central to cell growth, translation of milk protein genes, and activation of lipid regulatory factors), and MAPK (involved in proliferation and stress mitigation). Population genetic analysis further identified several SNPs in the *PRL* coding sequence.

PRL is a crucial regulatory hormone for mammary gland development and lactation in mammals, exhibiting significant stage-specific characteristics. During pregnancy, PRL promotes the proliferation and differentiation of mammary epithelial cells, facilitating mammary gland development. Post-parturition, it initiates milk biosynthesis by activating the JAK2-STAT5 signaling pathway (Freeman, et al., 2000). This study successfully cloned the *PRL* gene transcript from buffalo (*Bubalus bubalis*) mammary gland tissue, revealing a full-length CDS of 690 bp, encoding a polypeptide composed of 229 amino acid residues. The first 30 amino acids at the N-terminus of this protein form a signal peptide, and the subsequent 199 amino acids form the mature peptide, which is highly consistent with bovine PRL protein (Cao et al., 2002). Sequence alignment results showed that the CDS and transcriptional region structure of the buffalo *PRL* gene, along with the physicochemical characteristics, motifs, and domain composition of its encoded product, are highly consistent with those of other mammals, especially other Bovidae animals. Like its bovine counterpart, buffalo PRL contains a prolactin-like domain known to regulate endothelial cell proliferation via an autocrine mechanism (Clapp et al., 1998). This high degree of conservation provides a strong basis for investigating whether the molecular mechanisms of lactation are consistent between buffalo and other dairy species.

Our investigation into the expression patterns of the buffalo *PRL* gene confirmed its critical, stage-specific role in lactation. We observed that *PRL* mRNA abundance was significantly higher in mammary gland tissue during peak lactation compared to the dry period. This finding was further validated at the cellular level, where lactating BuMECs showed markedly higher *PRL* expression than non-lactating cells. This establishes PRL as a primary driver of lactation in buffalo, consistent with its established galactopoietic function in cows, goats, and sheep. Furthermore, this study corroborates the broad, multifunctional nature of PRL, as we detected its expression in numerous non-mammary tissues, including the heart, liver, spleen, lung, kidney, brain, cerebellum, rumen, small intestine, and muscle. The expression levels in these tissues also varied between lactation and non-lactation periods. For instance, the high expression of *PRL* in metabolically active organs like the liver and small intestine during lactation likely supports the mobilization of nutrients required for intensive milk synthesis (Aisaka, 1982). As a calcium-regulating hormone, PRL also enhances intestinal calcium absorption, ensuring an adequate supply for milk secretion (Charoenphandhu et al., 2010). These findings underscore that buffalo PRL orchestrates lactation not only through direct action on the mammary gland but also by systemically coordinating metabolic activities in other vital organs (Houdebine et al., 1985).

A cornerstone of this investigation is the demonstration that PRL acts as a potent mitogen for BuMECs. Overexpression of *PRL* significantly enhanced the proliferation and viability of BuMECs. This effect is mechanistically driven by the significant upregulation of key cell-cycle-promoting genes, including cyclin-dependent kinases (*CDK2*, *CDK4*) (Arnold and Papanikolaou, 2005) and their regulatory partners, *Cyclin D1* and *Cyclin E1* (Moiseeva et al., 2019; Sicinski et al., 1995). Since the population of secretory epithelial cells is a primary determinant of milk production capacity (Capuco et al., 2001), this mitogenic function of PRL is fundamental to establishing lactation potential. We further elucidated that this proliferative response is mediated by the coordinated activation of multiple signaling pathways. *PRL* overexpression significantly upregulated key components of the JAK2-STAT5 and PI3K-AKT-mTOR pathways. The JAK2-STAT5 pathway is the classic cascade essential for the differentiation and survival of mammary epithelial cells (Xin et al., 2020), while the PI3K-AKT-mTOR pathway is a central hub that integrates hormonal and nutritional signals to regulate cell growth (Costa-Mattioli and Monteggia, 2013; Jewell and Guan, 2013). Importantly, these pathways are interconnected: STAT5 can directly activate *Cyclin D1* transcription, and the PI3K-AKT-mTOR pathway also regulates *Cyclin D1* (Shi et al., 2017; Brockman et al., 2002; Brockman and Schuler, 2005). This indicates that PRL synergistically regulates the proliferation of BuMECs by participating in the JAK2-STAT5 pathway governing cell differentiation and modulating the PI3K-AKT-mTOR pathway controlling cell growth.

One of the most insightful findings of this study is the differential regulation of the mitogen-activated protein kinase (MAPK) pathway by PRL. The MAPK family, comprising primarily the ERK, JNK, and p38 subfamilies, transduces a wide array of extracellular signals to regulate proliferation, differentiation, and stress responses (Fanger et al., 1997; Lavoie and Therrien, 2015; McCain, 2013; Fata et al., 2007). We observed that *PRL* overexpression led to a significant increase in *ERK2* expression while simultaneously decreasing *JNK1* and *P38* expression. The upregulation of *ERK2* is consistent with PRL's mitogenic effects, as the ERK pathway is classically associated with growth-factor-induced proliferation (Lu and Xu, 2006). Conversely, the simultaneous downregulation of *JNK1* and *P38* is particularly noteworthy. These kinases are typically activated by cellular stressors and are often involved in pro-apoptotic signaling (Cuenda et al., 2007; Dhanasekaran and Reddy, 2008). Previous work has shown that in the absence of PRL, mammary cells exhibit a heightened stress response to inflammatory stimuli (Silva et al., 2017). Therefore, our observed pattern of MAPK regulation (activating *ERK2* while suppressing *JNK1*/*P38*) reveals a sophisticated dual function of PRL: it not only initiates proliferation but also actively protects the mammary epithelium from the physiological stress inherent to lactation.

This study confirmed that PRL powerfully promotes the synthesis of both buffalo milk protein and buffalo milk fat through a unified signaling hub. Overexpression of *PRL* in BuMECs led to a significant increase in the expression of casein genes (*CSN2*, *CSN3*) and a corresponding rise in casein content. Simultaneously, it upregulated the gene expression of key enzymes (*FASN*, *SCD*, *ACACA*) and core transcription factors (*PPARG*, *SREBF1*) for lipid synthesis, resulting in a significant accumulation of intracellular TAG. For milk protein synthesis, PRL activates the classic JAK2-STAT5 pathway, where phosphorylated STAT5 translocates to the nucleus to initiate casein gene transcription (Kelly et al., 2002; Kabotyanski et al., 2006; Murney et al., 2015). At the same time, activation of the PI3K-AKT-mTOR pathway is crucial for the transcription and translation of milk protein genes (Li et al., 2025), and PRL promotes milk protein synthesis by inducing the phosphorylation of mTOR and downstream effectors of the mTOR pathway (such as S6K1 and 4E-BP1) through the PI3K-AKT pathway (Bishop et al., 2006). For milk fat synthesis, our results show that these same pathways converge to regulate the master transcription factors SREBF1 and PPARG. Both mTOR and STAT5 are known to enhance the activity of these factors, thereby driving the expression of genes required for lipogenesis (Porstmann et al., 2008; Guo et al., 2019; McGillicuddy et al., 2009). This shows that PRL synergistically promotes milk protein and milk fat synthesis in BuMECs by participating in the JAK2-STAT5 pathway for casein gene and lipid factor transcription and by mediating the PI3K-AKT-mTOR pathway for milk protein gene translation and lipid factor activation. Moreover, the convergence of these pathways is further enhanced by their intrinsic crosstalk. Consistent with our observation that *PRL* overexpression simultaneously upregulates *STAT5A*/ B and *PI3K*/*AKT1*, prior studies demonstrate that activated STAT5 can transcriptionally upregulate PI3K/AKT1 (Creamer et al., 2010), while AKT1 enhances STAT5 activity by phosphorylating its co-activators (Rädler et al., 2017). This “JAK2-STAT5 → PI3K-AKT-mTOR → STAT5” interaction establishes a self-amplifying positive feedback loop that ensures a potent and sustained signal. Thus, this demonstrates that PRL, as the upstream signal, integrates the JAK2-STAT5 and PI3K-AKT-mTOR pathways to coordinately drive both the proliferation of BuMECs and milk component synthesis.

Genetic analysis of the buffalo *PRL* coding sequence identified five single-nucleotide polymorphisms (SNPs). Three of these (c.192G > A, c.427C > T, c.606C > T) were synonymous substitutions, while two (c.34C > T [p.R12C], c.430T > C [p.S144P]) were non-synonymous. Although predictive algorithms suggested these amino acid changes do not alter protein function, such SNPs can potentially influence mRNA stability or splicing, thereby affecting expression levels and milk production traits. This aligns with previous research in Italian Mediterranean river buffalo, where an SNP in the *PRL* gene was significantly associated with milk yield, protein, and fat content (Li et al., 2017). However, this differs from studies conducted in various dairy cow breeds. In dairy cows, multiple polymorphic sites within the *PRL* gene, particularly the RsaI restriction site polymorphism located in exon 3, have been repeatedly shown to be significantly associated with core economic traits such as milk yield, milk fat percentage, and milk protein percentage (Dybus et al., 2005). In Romanian Brown cattle, the AA genotype at the rs211032652 locus is significantly associated with higher milk fat and protein content (Ilie et al., 2023). This suggests that the genetic basis for lactation trait variation may differ between buffalo and cattle. The observed deviation from the Hardy–Weinberg equilibrium at the c.430T > C locus in the swamp buffalo population may be attributed to several potential factors. These include artificial selection pressure, population bottleneck effects, and non-random mating patterns. The specific mechanism underlying this deviation requires further investigation with expanded population sampling and genome-wide association analysis to confirm. In addition, the nucleotide difference sites in the *PRL* CDS between buffalo and other Bovidae animals can serve as molecular markers to distinguish buffalo from other Bovidae animals. Notably, although PRL proteins in buffalo and cattle are highly homologous, they exhibit notable differences when compared with the more distantly related *Ovis* genus. Specifically, the difference sites c.19, c.413, and c.583 between buffalo and *Ovis* species resulted in different encoded amino acids, which may have potential effects on the structure and function of PRL protein.

Our findings reveal that the expression regulation of the buffalo *PRL* gene is tightly coupled with its lactation function and mammary gland development. Its gene structure, function, and core regulatory mechanisms are highly similar to those of other bovine species, and the core signaling pathways governing milk component synthesis also exhibit a high degree of evolutionary conservation among mammals. However, this conserved regulatory framework stands in stark contrast to the significant differences in lactation phenotypes between buffalo (e.g., low milk yield, fewer mammary alveoli) and high-yielding dairy cattle. This raises a key scientific question: given the conservation of the core regulatory mechanisms, what is the molecular basis for the divergence in lactation performance between species? This study posits that this phenotypic divergence does not stem from a qualitative difference in the regulatory mechanism itself, but rather from quantitative differences in regulatory efficiency and genetic background. To this end, we propose the “Regulatory Element Hypothesis” as a core explanation: although the regulatory principles are conserved, critical sequence variations may exist in the non-coding regulatory elements (e.g., promoters, enhancers) of the *PRL* gene between buffalo and cattle. In the bovine, the expression of *PRL* depends on the 5^′^-flanking region sequence; variations in this region may alter transcriptional factor binding sites and affect *PRL* expression (Brym et al., 2007; Zhang et al., 1994; Hart et al., 1993). It is therefore plausible that similar variations, particularly those affecting the binding sites of key transcription factors such as POU1F1, could alter the transcriptional efficiency and dynamics of the *PRL* gene, ultimately leading to species-specific differences in hormone levels and milk production. Furthermore, our study found that the association patterns between *PRL* gene polymorphisms and production traits differ distinctly between buffalo and dairy cattle. Unlike in dairy cattle, where multiple functional SNPs have been reported, the variations identified in the coding region of the buffalo *PRL* gene in this study were all functionally neutral. This lends further support to the inference that the decisive differences likely reside in the non-coding regulatory regions. In conclusion, we postulate that when an evolutionarily conserved molecular regulatory framework operates upon different genetic backgrounds and anatomical foundations (such as the inherently lower number of mammary alveoli in buffalo), subtle genetic differences in its regulatory efficiency can be amplified, leading to the significant divergence of lactation phenotypes observed between species.

Despite these findings, this study has limitations. First, the genetic analysis focused only on coding-region polymorphisms of the buffalo *PRL* gene, neglecting non-coding regulatory elements (e.g., promoters, enhancers) that may be critical for transcriptional regulation. Second, although functional validation using BuMECs provided useful insights, the in vitro model cannot fully replicate in vivo complexity. The lack of in vivo evidence, such as from transgenic or gene-edited animals, limits extrapolation to the whole organism. Future studies could employ whole-genome resequencing to examine variation across both coding and non-coding regions of the *PRL* gene. In addition, applying transgenic overexpression or CRISPR/Cas9-mediated knockout in animal models would strengthen in vivo functional validation. These approaches would enhance the understanding of PRL's role in buffalo lactation and support genetic strategies for improving milk production.

## 5 Conclusions

This study comprehensively characterizes the buffalo prolactin (*PRL*) gene, confirming its high conservation among Bovidae and its pivotal role in lactation. We demonstrate that *PRL* expression is significantly upregulated in the mammary gland during lactation and that it functions as a potent mitogen for buffalo mammary epithelial cells (BuMECs). This proliferative effect is driven by the coordinated activation of the JAK2-STAT5, PI3K-AKT-mTOR, and MAPK signaling pathways. Furthermore, we establish that PRL is a key driver of milk biosynthesis, stimulating both casein and fat synthesis through a unified signaling network involving the JAK2-STAT5 and PI3K-AKT-mTOR pathways. While genetic analysis identified five SNPs in the coding sequence, the non-synonymous variations were not predicted to alter protein function. Collectively, this research provides a detailed molecular framework of PRL's function in buffalo and offers a crucial theoretical foundation for developing genetic strategies aimed at enhancing lactation performance and milk yield in this important dairy species.

## Supplement

10.5194/aab-68-703-2025-supplement The supplement related to this article is available online at https://doi.org/10.5194/aab-68-703-2025-supplement.

## Data Availability

The datasets generated are available from the corresponding author on request.
