# Supplementary material for: Molecular characterization and functional analysis of buffalo prolactin (PRL) in mammary gland development and lactation
Source: Arch Anim Breed. 2025 Dec 3;68(4):703–19. doi: 10.5194/aab-68-703-2025 (PMC13524326; doi:10.5194/aab-68-703-2025)
Supplement: Supplementary file 1 [file aab-68-703-2025-supplement.pdf]

Supplement of Arch. Anim. Breed., 68, 703–719, 2025
https://doi.org/10.5194/aab-68-703-2025-supplement
© Author(s) 2025. CC BY 4.0 License.

Supplement of

Molecular characterization and functional analysis of buffalo prolactin (PRL) in mammary gland development and lactation

Lige Huang et al.

Correspondence to: Zhipeng Bao (baopeng976@163.com) and Yongwang Miao (miaoyw1@ynau.edu.cn)

The copyright of individual parts of the supplement might differ from the article licence.

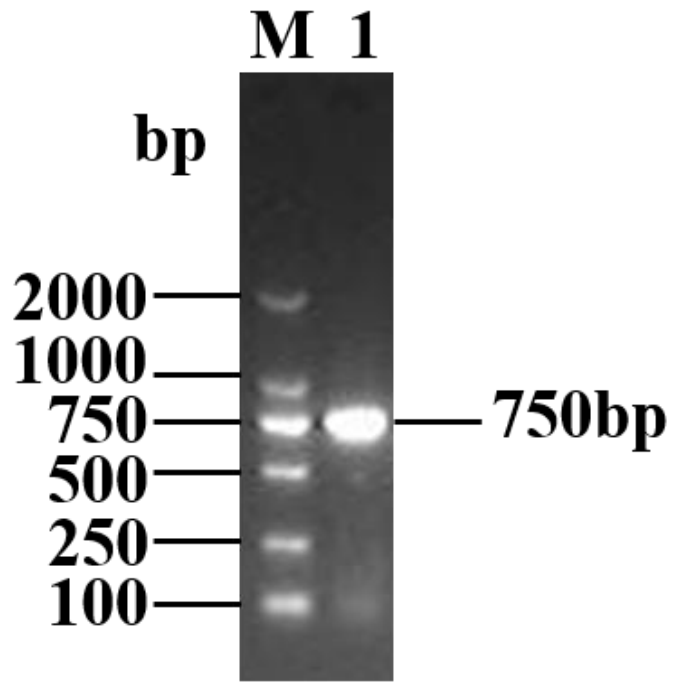

Figure S1. PCR amplicon of the buffalo *PRL* gene. M, DL2000 marker; 1, PCR product.

```
1   ATGGACAGCAAAGGTTCGTCACAGAAAGGGTCCCGCCTGCTCCTGCTGCTGGTGGTGTCAAATCTACTCTTGTGCCAGGGTGTGGTCTCC 90
1   M D S K G S S Q K G S R L L L L L V V S N L L L C Q G V V S 30
91  ACCCCCGTCTGTCCCAATGGGCCTGGCAACTGCCAGGTGTCCCTTCGAGACCTGTTTGACCGGGCAGTCATGGTGTCCCACTACATCCAT 180
31  T P V C P N G P G N C Q V S L R D L F D R A V M V S H Y I H 60
181 GACCTCTCCTCGGAAATGTTCAACGAATTTGATAAACGGTATGCCCAGGGCAAAGGGTTCATTACCATGGCCCTCAACAGCTGCCATACC 270
61  D L S S E M F N E F D K R Y A Q G K G F I T M A L N S C H T 90
271 TCCTCCCTTCCTACCCCTGAAGACAAAGAACAAGCCCAACAGACCCACCATGAAGTCCTTATGAGCTTGATTCTTGGGTTGCTGCGCTCC 360
121 S S L P T P E D K E Q A Q Q T H H E V L M S L I L G L L R S 120
361 TGGAATGACCCTCTGTATCACCTAGTCACAGAGGTGCGGGGTATGAAAGGAGCCCCAGATGCTATCCTATCGAGGGCCATAGAGATTGAG 450
151 W N D P L Y H L V T E V R G M K G A P D A I L S R A I E I E 150
451 GAAGAAAACAAACGACTTCTGGAAGGCATGGAGATGATATTTGGCCAGGTTATTCCTGGAGCCAAAGAGACTGAGCCCTACCCTGTGTGG 540
181 E E N K R L L E G M E M I F G Q V I P G A K E T E P Y P V W 180
541 TCAGGACTCCCATCCCTGCAAACTAAGGATGAAGAGGCGCGTTATTCTGCTTTTTATAACCTGCTCCACTGCCTGCGCAGGGATTCAAGC 630
211 S G L P S L Q T K D E E A R Y S A F Y N L L H C L R R D S S 210
631 AAGATTGACACTTACCTTAAGCTCCTGAATTGCAGAATCATCTACAACAACAACTGCTAA 690
241 K I D T Y L K L L N C R I I Y N N N C * 229
```

Figure S2. CDS and encoded amino acid sequence of buffalo *PRL*. The shaded region represents the PRL conserved domain (AA32-228); "*" indicates the stop codon.

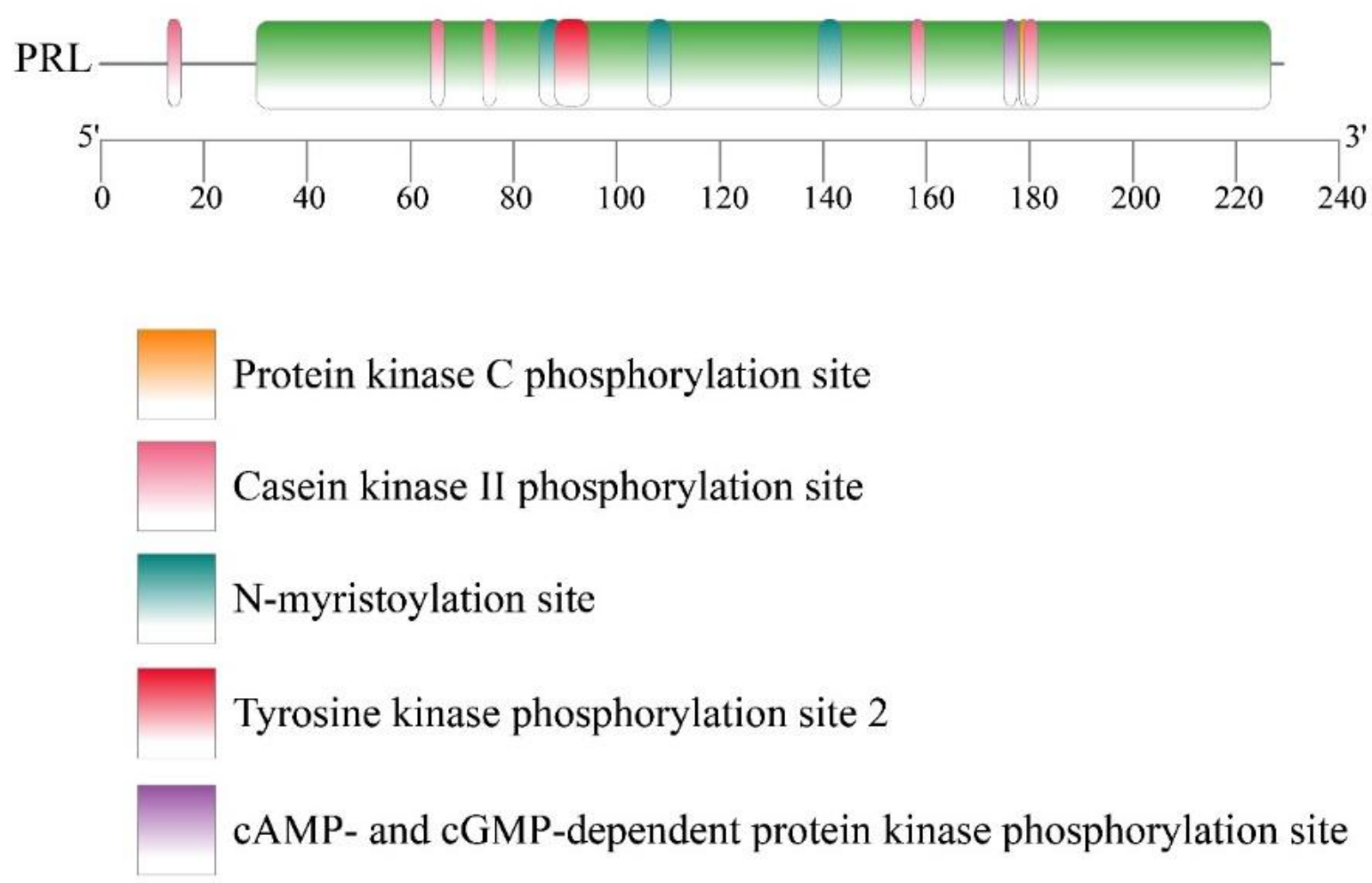

Figure S3. Prediction of functional modification sites of buffalo PRL.

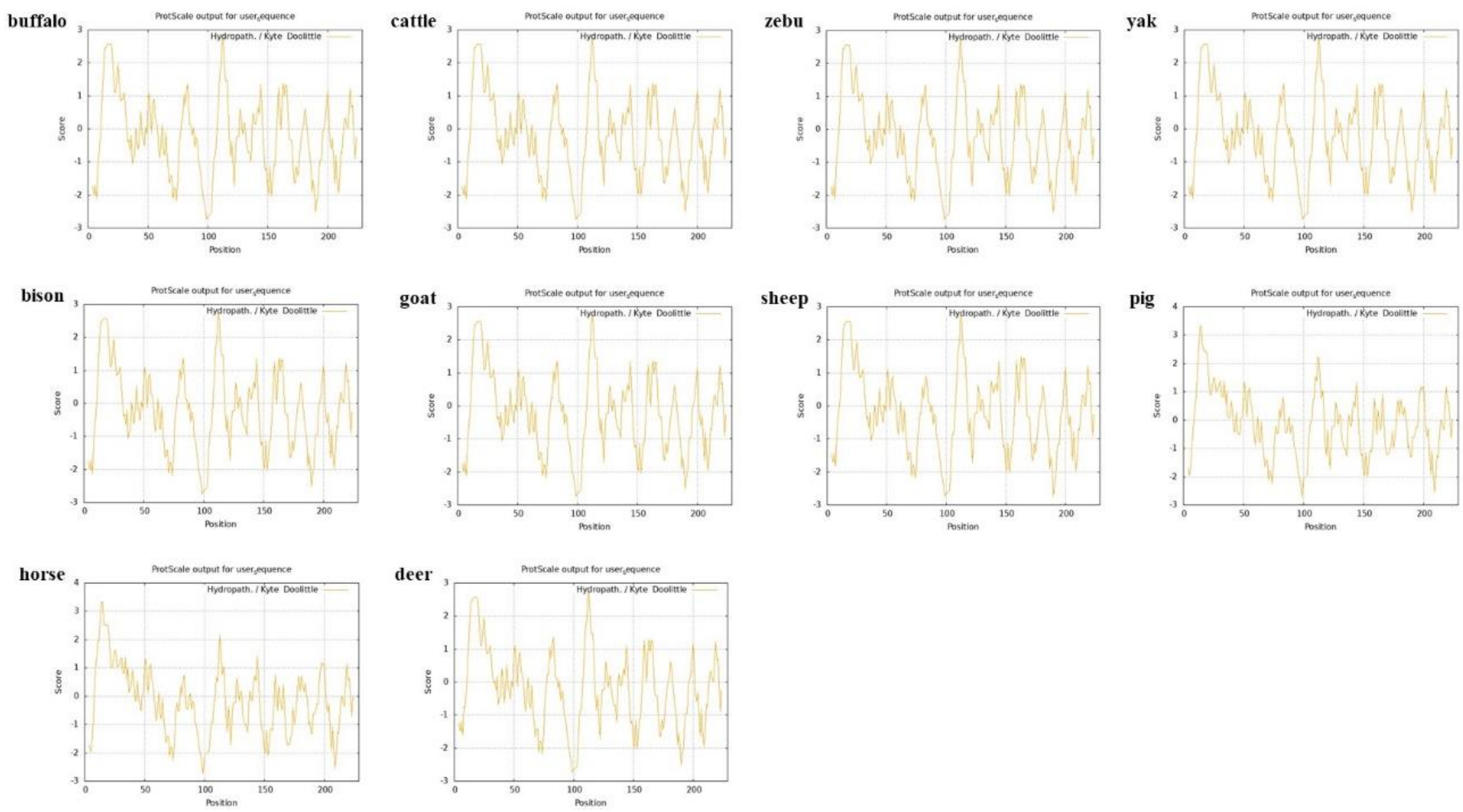

Figure S4. Predicted hydrophilicity of the PRL protein across different mammalian species.

buffalo

```
         10        20        30        40        50        60        70
          |         |         |         |         |         |         |
MDSKGSSQKGSRLLLLLVVSNLLLCQGVVSTPVCPNGPGNCQVSLRDLFDRAVMVSHYIHDLSSEMFNEF
cccccccccchhheeeehhhhhhhccccccccccccccccccchhhhhhhhhhhhhhhhhhhhhhhhhhh
DKRYAQGKGFITMALNSCHTSSLPTPEDKEQAQQTHHEVLMSLILGLLRSWNDPLYHLVTEVRGMKGAPD
hhhhhttcchhhcccccccccccccccchhhhhcchhhhhhhhhhhhhhhhcchhhhhhhhhhhcttcch
AILSRAIEIEEENKRLLEGMEMIFGQVIPGAKETEPYPVWSGLPSLQTKDEEARYSAFYNLLHCLRRDSS
hhhhhhhhhhhhhhhhhhhhhhhhhhhcccccccccccccccccccccchhhhhhhhhhhhhhhhccccc
KIDTYLKLLNCRIIYNNNC
hhhhhhhhhhhhhcccccc
```

cattle

```
         10        20        30        40        50        60        70
          |         |         |         |         |         |         |
MDSKGSSQKGSRLLLLLVVSNLLLCQGVVSTPVCPNGPGNCQVSLRDLFDRAVMVSHYIHDLSSEMFNEF
ecccccccttchheeeehhhhhhhccccccccccccccccccchhhhhhhhhhhhhhhhhhhhhhhhhhh
DKRYAQGKGFITMALNSCHTSSLPTPEDKEQAQQTHHEVLMSLILGLLRSWNDPLYHLVTEVRGMKGAPD
hhhhhtcchhhhhccccccccccccccchhhhhhhhhhhhhhhhhhhhhhhcchhhhhhhhhhhcttcch
AILSRAIEIEEENKRLLEGMEMIFGQVIPGAKETEPYPVWSGLPSLQTKDEDARYSAFYNLLHCLRRDSS
hhhhhhhhhhhhhhhhhhhhhhhhhhhcccccccccccccccccchhccchhhhhhhhhhhhhhhhccccc
KIDTYLKLLNCRIIYNNNC
hhhhhhhhhhhhhccttcc
```

zebu

```
         10        20        30        40        50        60        70
          |         |         |         |         |         |         |
MDSKGSSQKGSRLLLLLVVSNLLLCQGVVSTPVCPNGPGNCQVSLRDLFDRAVMVSHYIHDLSSEMFNEF
ecccccccttchheeeehhhhhhhccccccccccccccccccchhhhhhhhhhhhhhhhhhhhhhhhhhh
DKRYAQGKGFITMALNSCHTSSLPTPEDKEQAQQTHHEVLMSLILGLLRSWNDPLYHLVTEVRGMKGAPD
hhhhhtcchhhhhccccccccccccccchhhhhhhhhhhhhhhhhhhhhhhcchhhhhhhhhhhcttcch
AILSRAIEIEEENKRLLEGMEMIFGQVIPGAKETEPYPVWSGLPSLQTKDEDARYSAFYNLLHCLRRDSS
hhhhhhhhhhhhhhhhhhhhhhhhhhhcccccccccccccccccchhccchhhhhhhhhhhhhhhhccccc
KIDTYLKLLNCRIIYNNNC
hhhhhhhhhhhhhccttcc
```

yak

```
         10        20        30        40        50        60        70
          |         |         |         |         |         |         |
MDSKGSSQKGSRLLLLLVVSNLLLCQGVVSTPVCPNGPGNCQVSLRDLFDRAVMVSHYIHDLSSEMFNEF
ecccccccttchheeeehhhhhhhccccccccccccccccccchhhhhhhhhhhhhhhhhhhhhhhhhhh
DKRYAQGKGFITMALNSCHTSSLPTPEDKEQAQQTHHEVLMSLILGLLRSWNDPLYHLVTEVRGMKGAPD
hhhhhtcchhhhhccccccccccccccchhhhhhhhhhhhhhhhhhhhhhhcchhhhhhhhhhhcttcch
AILSRAIEIEEENKRLLEGMEMIFGQVIPGAKETEPYPVWSGLPSLQTKDEDARYSAFYNLLHCLRRDSS
hhhhhhhhhhhhhhhhhhhhhhhhhhhcccccccccccccccccchhccchhhhhhhhhhhhhhhhccccc
KIDTYLKLLNCRIIYNNNC
hhhhhhhhhhhhhccttcc
```

bison

```
         10        20        30        40        50        60        70
          |         |         |         |         |         |         |
MDSKGSSQKGSRLLLLLVVSNLLLCQGVVSTPVCPNGPGNCQVSLRDLFDRAVMVSHYIHDLSSEMFNEF
ecccccccttchheeeehhhhhhhccccccccccccccccccchhhhhhhhhhhhhhhhhhhhhhhhhhh
DKRYAQGKGFITMALNSCHTSSLPTPEDKEQAQQTHHEVLMSLILGLLRSWNDPLYHLVTEVRGMKGAPD
hhhhhtcchhhhhccccccccccccccchhhhhhhhhhhhhhhhhhhhhhhcchhhhhhhhhhhcttcch
AILSRAIEIEEENKRLLEGMEMIFGQVIPGAKETEPYPVWSGLPSLQTKDEDARYSAFYNLLHCLRRDSS
hhhhhhhhhhhhhhhhhhhhhhhhhhhcccccccccccccccccchhccchhhhhhhhhhhhhhhhccccc
KIDTYLKLLNCRIIYNNNC
hhhhhhhhhhhhhccttcc
```

goat

```
         10        20        30        40        50        60        70
          |         |         |         |         |         |         |
MDSKGSAQKGSRLLLLLVVSNLLLCQGVVSTPVCPNGPGNCQVSLRDLFDRAVMVSHYIHNLSSEMFNEF
ecccccchhtthhhheehhhhhhhccttcccccccccccccccchhhhhhhhhhhhhhhhhhhhhhhhhhh
DKRYAQGKGYITMALNSCHTSSLPTPEDKEQAQQTHHEVLMSLILGLLRSWNDPLYHLVTEVRGMKGVPD
hhhhcccccccccccccccccccccccchhhhhhhhhhhhhhhhhhhhhhhcchhhhhhhhhhhcttcch
AILSRAIEIEEENKRLLEGMEMILGQVIPGAKETEPYPVWSGLPSLQTKDEEARHSAFYNLLHCLRRDSS
hhhhhhhhhhhhhhhhhhhhhhhhhhhcccccccccccccccccccccchhhhhhhhhhhhhhhhccccc
KIDTYLKLLNCRIIYNNNC
hhhhhhhhhhhhhhettcc
```

sheep

```
         10        20        30        40        50        60        70
          |         |         |         |         |         |         |
MVVMISRNTLHQDSKGSAQKGSRLLLLLVVSNLLLCQGVVSTPVCPNGPGDCQVSLRDLFDRAVMVSHYI
eeeeecttceehcccccccctthhheeeehhhhhhcttcccccccccccccchhhhhhhhhhhhhhhhhhh
HNLSSEMFNEFDKRYAQGKGFITMALNSCHTSSLPTPEDKEQAQQTHHEVLMSLILGLLRSWNDPLYHLV
hhhhhhhhhhhhhhhccccchhhcccccccccccccccccchhhhhhhhhhhhhhhhhhhhhhhcchhhhh
TEVRGMKGVPDAILSRAIEIEEENKRLLEGMEMIFGQVIPGAKETEPYPVWSGLPSLQTKDEDARHSAFY
hhhhhcccchhhhhhhhhhhhhhhhhhhhhhhhhhhhcccccccccccchhhcchhccccccchhhhhhhh
NLLHCLRRDSSKIDTYLKLLNCRIIYNNNC
hhhhhhcccchhhhhhhhhhhhhhccttcc
```

pig

```
         10        20        30        40        50        60        70
          |         |         |         |         |         |         |
MVVMISRNTLHQDSKGSAQKGSRLLLLLVVSNLLLCQGVVSTPVCPNGPGDCQVSLRDLFDRAVMVSHYI
eeeeecttceehcccccccctthhheeeehhhhhhcttcccccccccccccchhhhhhhhhhhhhhhhhhh
HNLSSEMFNEFDKRYAQGKGFITMALNSCHTSSLPTPEDKEQAQQTHHEVLMSLILGLLRSWNDPLYHLV
hhhhhhhhhhhhhhhccccchhhcccccccccccccccccchhhhhhhhhhhhhhhhhhhhhhhcchhhhh
TEVRGMKGVPDAILSRAIEIEEENKRLLEGMEMIFGQVIPGAKETEPYPVWSGLPSLQTKDEDARHSAFY
hhhhhcccchhhhhhhhhhhhhhhhhhhhhhhhhhhhcccccccccccchhhcchhccccccchhhhhhhh
NLLHCLRRDSSKIDTYLKLLNCRIIYNNNC
hhhhhhcccchhhhhhhhhhhhhhccttcc
```

horse

```
         10        20        30        40        50        60        70
          |         |         |         |         |         |         |
MDSKGSSQKGSRLLLLLVVSNLLLCQGVVSTPVCPNGPGNCQVSLRDLFDRAVMVSHYIHDLSSEMFNEF
ecccccccttchheeeehhhhhhhccccccccccccccccccchhhhhhhhhhhhhhhhhhhhhhhhhhh
DKRYAQGKGFITMALNSCHTSSLPTPEDKEQAQQTHHEVLMSLILGLLRSWNDPLYHLVTEVRGMKGAPD
hhhhhtcchhhhhccccccccccccccchhhhhhhhhhhhhhhhhhhhhhhcchhhhhhhhhhhcttcch
AILSRAIEIEEENKRLLEGMEMIFGQVIPGAKETEPYPVWSGLPSLQTKDEDARYSAFYNLLHCLRRDSS
hhhhhhhhhhhhhhhhhhhhhhhhhhhcccccccccccccccccchhccchhhhhhhhhhhhhhhhccccc
KIDTYLKLLNCRIIYNNNC
hhhhhhhhhhhhhccttcc
```

deer

```
         10        20        30        40        50        60        70
          |         |         |         |         |         |         |
MDSKGSAQKGSRLLLLLVVSNLLLCQGVVSTPVCPNGPGNCQVSLRDLFDRAVMVSHYIHNLSSEMFNEF
ecccccchhtthhhheehhhhhhhccttcccccccccccccccchhhhhhhhhhhhhhhhhhhhhhhhhhh
DKRYAQGKGYITMALNSCHTSSLPTPEDKEQAQQTHHEVLMSLILGLLRSWNDPLYHLVTEVRGMKGVPD
hhhhcccccccccccccccccccccccchhhhhhhhhhhhhhhhhhhhhhhcchhhhhhhhhhhcttcch
AILSRAIEIEEENKRLLEGMEMILGQVIPGAKETEPYPVWSGLPSLQTKDEEARHSAFYNLLHCLRRDSS
hhhhhhhhhhhhhhhhhhhhhhhhhhhcccccccccccccccccccccchhhhhhhhhhhhhhhhccccc
KIDTYLKLLNCRIIYNNNC
hhhhhhhhhhhhhhettcc
```

Figure S5. Secondary structure analysis of PRL proteins from buffalo and its mammalian orthologs.

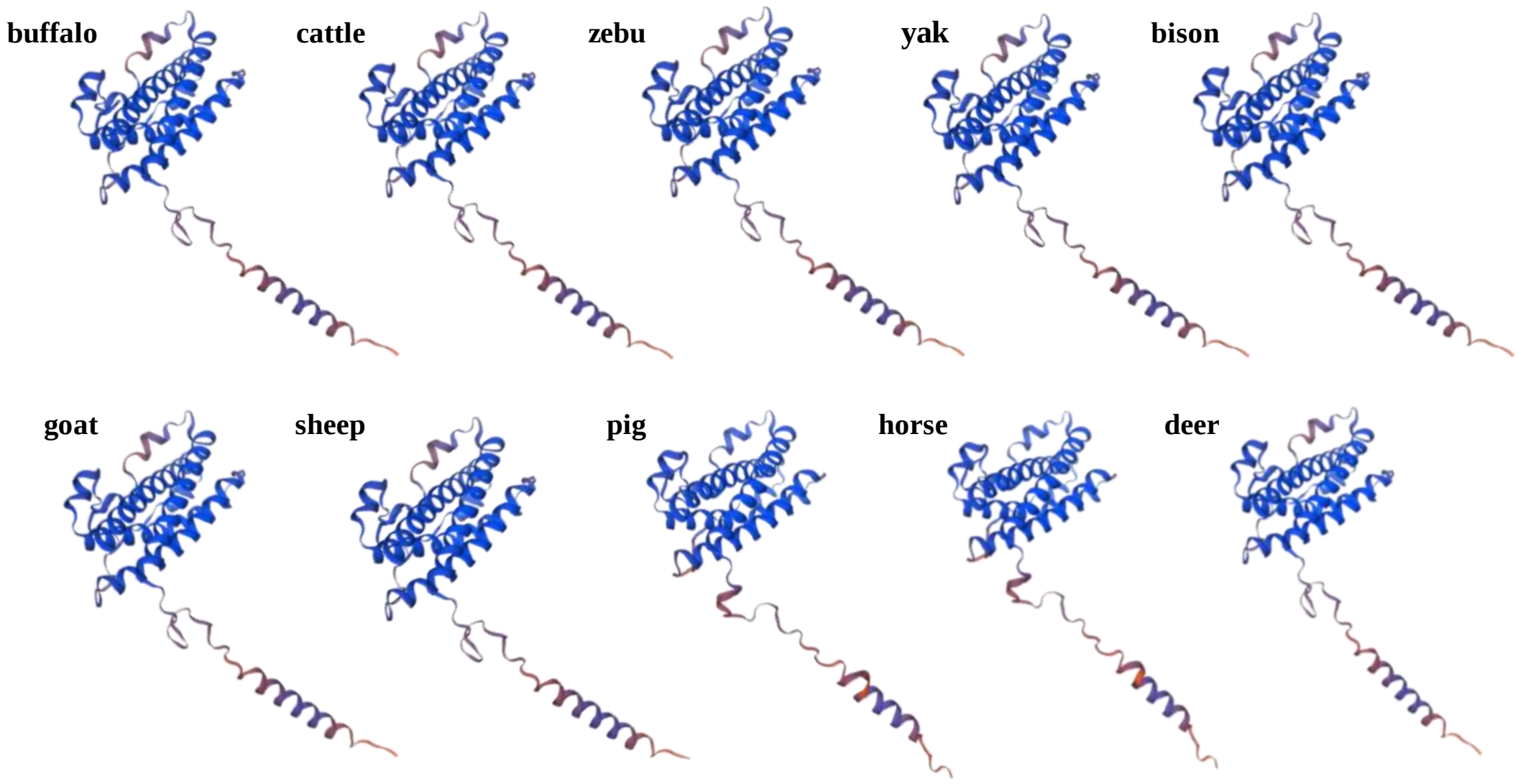

Figure S6. Predicted three-dimensional structures of PRL from buffalo and other mammalian species.

```
                            11111111
                      1346800346999
                     7 2201016844256
B1                   S RPNDFQHASFEYS
B2                   .?........P....
B3                   .?.............
B4                   .?........P....
B5                   .?........P....
B6                   .?.............
B7                   .?........P....
B8                   .?C............
B9                   .?C............
B10                  .?C.......P....
Buffalo_NP_001277814 .?........P....
Buffalo_XP_006072923 .AC............
Cattle_NP_776378     .?..........D..
Bison_XP_010843869   .?..........D..
Yak_XP_014333241     .A..........D..
Zebu_XP_019841745    .?.?........D.?
Sheep_CAA31839       A?...N...V..DH.
Goat_NP_001272476    A?...NY..V.L.H.
```

Figure S7. Amino acid difference sites of buffalo PRL haplotypes and PRL in other Bovidae species.

Table S1. Primer information used in this study.

Primer	Template for primers	Primer sequence (5'→3')*	Product length (bp)	Annealing temperature (°C)	Amplification efficiency	Purpose
PRL	EF054878	F: TTCCTGGTGAAGTGTGTTTCT<br>R: AATGTGGGCTTAGCAGTTG	736	60	/	CDS isolation
PRL	OL960754	F: ATGGACAGCAAAGGTTCGT<br>R: GAACATTTCCGAGGAGAGG	201	58.8	1.96	Differential expression
ACTB	NM_001290932	F: TCTTGGTCACCTCGTCGTC<br>R: GGCGCGATGATCTTGAT	196	60	1.87	Differential expression
GAPDH	XM_006065800	F: ATGGAGAAGGCTGGGGCTCA<br>R: GCAGGAGGCATTGCTGACAA	144	60	1.90	Differential expression
RPS23	XM_006059350	F: ACCGACGAGACCAGAAGT<br>R: CTCCAGGAATGTCACCAA	306	60	1.85	Differential expression
*PRL*_Exon1	NC_059158	F: TCTCAATGACGGAAATA<br>R: GATAAAACCCAACAACTC	224	50	/	SNP detection
*PRL*_Exon2	NC_059158	F: ATCTTAGAGCGGCTCATG<br>R: CACTTACG AAAGGTTTC	265	54	/	SNP detection
*PRL*_Exon3	NC_059158	F: GACAAGCAACTGTTTTCA<br>R: CATAATGTTTCTCCACCT	411	54	/	SNP detection

*PRL*_Exon4	NC_059158	F: CAAAGTATAAACCGATAG<br>R: GCTGGTGGAATTCTAAT	554	51	/	SNP detection
*PRL*_Exon5	NC_059158	F: CTTTTGGATGTTTAGGTT<br>R: TCTCAGAAATGGATAGGA	236	50	/	SNP detection
PRL	OL960754	F: AAGCTTATGGACAGCAAAGGTTCGTC<br>R: GAATTCGCAGTTGTTGTTGTAGATGA	699	59	/	Gene overexpression
PI3K	XM_006069974	F: CAGTCACCTCTCAACCCA<br>R: GCCGTAAATCATCACCAT	160	60	1.98	Expression level detection
AKT	NM_001290841	F: CCAAGTCCCTGCTCTCGG<br>R: TTCGCTGTCCACCCCCTC	266	60	1.89	Expression level detection
mTOR	XM_025285881	F: ATGCTGTCCCTGGTCCTTATG<br>R: GGGTCAGAGAGTGGCCTTCAA	178	60	1.87	Expression level detection
EIF4E	NM_001319800	F: GATGGGCACTCTGGTTTT<br>R: CCTCCTCGTTTGTTTTTC	208	60	1.88	Expression level detection
S6K1	XM_006065441	F: TGCTGCTTCTCGTCTTGG<br>R: GGTTGGCACTTTCACTGA	224	60	1.99	Expression level detection
PRLR	XM_025270634	F: CCACCCACCACGACTGATGTAAA<br>R: ATGACAGCAGAAAGGACGG	287	60	1.94	Expression level detection
JAK2	XM_006068203	F: CGCTGGTGGGCTTTTACT<br>R: CATCTCTTTCTGCCATTTCA	252	60	1.86	Expression level detection
STAT5A	XM_006055752	F: GCTTGGGACTCAATAGAT<br>R: CAGCCTCTGTTCATTGTA	191	60	1.95	Expression level detection
STAT5B	XM_025280122	F: AAGCCTGATGGCACCTTCT<br>R: CGGTCGGGAAACACGTAGA	236	60	1.97	Expression level detection
CDK2	XM_006075063	F: TCATCGAGTCCTGCACCGAGA<br>R: AACCAAGGCTCCATATGTCC	212	57	1.87	Expression level detection
CDK4	XM_006077918	F: TGCCAACTGCATCGTTCACC<br>R: GATACAGCCAACGCTCCAC	214	57	1.89	Expression level detection
CCNE1	XM_025268989	F: AGCCTAAAATGCGAGCAA<br>R: CATAAGCAAACTGGTGCAAC	225	53.5	1.91	Expression level detection
CCND1	XM_006072357	F: CCAACCTCCTCAACGACCGAGT<br>R: TTTTCACGGGCTCCAGCGACA	228	61	1.95	Expression level detection
ERK2	XM_025267233	F: GGAAGCATTACCTCGACCAG<br>R: CTGCTCCACCTCAATCCTC	230	58	1.92	Expression level detection
JNK1	XM_025284904	F: CTGGAATTATTCACCGGGAC<br>R: ACAGACCATAAATCCACGTT	199	57	1.89	Expression level detection
P38	XM_006050391	F: AGCTTACAGATGACCACGTT<br>R: CATCTGTATGTCGAGCCAGT	168	58.5	1.90	Expression level detection
CSN2	NM_001290879	F: GGCTCCTAAGCACAAAGA<br>R: AGGGGGAAACATGACAGT	172	60	1.99	Expression level detection
CSN3	NM_001290972	F: TTTACCTTGGGTGTTCCT<br>R: TTTCTCACAGCGTATTGG	184	60	1.96	Expression level detection
SREBF1	XM_025280442	F: GCACCGAGGCCAAGTTGAATAA<br>R: CAGGTCCTTCAGCGATTTGCTT	146	57	1.84	Expression level detection
PPARG	NM_001290893	F: GCTCCAAGAGTACCAAAGTG<br>R: GTCCTCCTGAAGAAACCCTT	204	53.7	1.91	Expression level detection
FASN	XM_006061793	F: AGGCCAGCTCCGAAGGCAACA<br>R: TACCACGTCGGCCACTTGTGTC	209	64.3	1.93	Expression level detection
ACACA	XM_025281124	F: CCTCTTCAGACAGGTTCAAGC<br>R: TTCACCGCACACTGTTCCA	234	55	1.88	Expression level detection
SCD	NM_001290915	F: CGTGCCGTGGTATCTGTGG<br>R: AAAGGTGTGGTGGTAGTTGTGG	217	56.2	1.92	Expression level detection

***,** F represents upstream primer, R represents downstream primer; The underlined bases indicate the enzyme cleavage sites.

Table S2. List of PRL sequences retrieved from the NCBI database.

**Species**	**Accession number of nucleotide sequences**	**Accession number of protein sequences**	**CDS length (bp)**
Buffalo (this study)	OL960754.1	UZN72311.1	690
Buffalo	NM_001290885.1	NP_001277814.1	690
Buffalo_X1	XM_006072861.4	XP_006072923.3	693
Cattle	NM_173953.2	NP_776378.2	690

Zebu	XM_019986186.1	XP_019841745.1	690
Yak	XM_005894272.2	XP_005894334.1	690
Bison	XM_010845567.1	XP_010843869.1	690
Goat	NM_001285547.1	NP_001272476.1	690
Sheep	NM_001009306.1	NP_001009306.1	723
Pig	NM_213926.1	NP_999091.1	690
Horse	NM_001081896.1	NP_001075365.1	690
Deer	XM_043908398.1	XP_043764333.1	690
Mouse	NM_011164.2	NP_035294.2	687

Table S3. Physicochemical properties of PRL from buffalo and other mammalian species.

**Basic physicochemical properties**	**Buffalo (this study)**	**Cattle**	**Zebu**	**Yak**	**Bison**	**Goat**	**Sheep**	**Pig**	**Horse**	**Deer**
Number of amino acids	229	229	229	229	229	229	240	229	229	229
molecular weight (kD)	25.81	25.79	25.79	25.79	25.79	25.79	27.06	26.12	25.27	25.79
Isoelectric point (PI)	5.98	5.98	5.98	5.98	5.98	6.24	6.30	6.30	6.71	6.16
Negatively charged residues (Asp+Glu)	27	27	27	27	27	26	27	27	29	26
Positively charged residues (Arg+Lys)	23	23	23	23	23	23	24	24	28	23
Hydrophobic amino acids (A, I, L, F, W, V)	73	73	73	73	73	73	78	81	82	74
Polar amino acids (N, C, Q, S, T, Y)	66	66	66	66	66	66	68	65	62	67
Instability index (II)	56.35	55.77	55.77	55.77	55.77	57.72	56.76	56.24	60.27	57.03
Grand average of hydropathicity	-0.246	-0.246	-0.246	-0.246	-0.246	-0.246	-0.212	-0.165	-0.208	-0.224
Aliphatic index (AI)	92.36	92.36	92.36	92.36	92.36	95.33	95.00	103.45	105.98	93.62

Table S4. Predicted secondary structure composition of buffalo PRL and its orthologs.

**Structer**	**Buffalo**	**Cattle**	**Zebu**	**Yak**	**Zebu**	**Goat**	**Sheep**	**Pig**	**Horse**	**Deer**
Random coil (%)	39.74	35.37	35.73	35.73	35.73	36.68	34.17	35.73	35.73	34.17
Alpha helix (%)	56.77	59.39	59.39	59.39	59.39	58.08	57.92	59.39	59.39	57.92
Beta turn (%)	1.75	3.06	3.06	3.06	3.06	3.49	3.33	3.06	3.06	3.33
Extended strand (%)	1.75	2.18	2.18	2.18	2.18	1.75	4.58	2.18	2.18	4.58

Table S5. Analysis of Relative Synonymous Codon Usage for the buffalo *PRL* gene.

Amino acid/Codon	Ser			Leu					
	UCG	→	UCA	CUA	→	UUA	CUC	→	CUU
Buffalo (Reference sequence)	0.60		1.20	0.58		0.00	1.35		1.16
Buffalo (SNP192)	0.30		1.50	0.58		0.00	1.35		1.16
Buffalo (SNP427)	0.60		1.20	0.39		0.19	1.35		1.16
Buffalo (SNP606)	0.60		1.20	0.58		0.00	1.16		1.35

Table S6. Haplotype frequencies for the *PRL* gene in the studied buffalo population.

	**Haplotype**	**Actual frequency**	**Expected frequency**
B1	CGCTC	0.568831	0.565762
B2	CGCCT	0.015584	0.014780
B3	CGTTC	0.109091	0.112134
B4	CGTCT	0.001299	0.000834
B5	CACCC	0.014286	0.017186
B6	CATTC	0.080519	0.080403
B7	CATCC	0.011688	0.008762
B8	TGCTC	0.090909	0.090779
B9	TGCTT	0.057143	0.056004
B10	TGCCT	0.050649	0.051323
